# Transcription Factor SomA Is Required for Adhesion, Development and Virulence of the Human Pathogen *Aspergillus fumigatus*

**DOI:** 10.1371/journal.ppat.1005205

**Published:** 2015-11-03

**Authors:** Chi-Jan Lin, Christoph Sasse, Jennifer Gerke, Oliver Valerius, Henriette Irmer, Holm Frauendorf, Thorsten Heinekamp, Maria Straßburger, Van Tuan Tran, Britta Herzog, Susanna A. Braus-Stromeyer, Gerhard H. Braus

**Affiliations:** 1 Department of Molecular Microbiology and Genetics, Institute of Microbiology & Genetics, Georg-August-University Göttingen, Göttingen, Germany; 2 Institute for Organic and Biomolecular Chemsitry, Georg-August-University Göttingen, Göttingen, Germany; 3 Molecular and Applied Microbiology, Leibniz Institute for Natural Product Research and Infection Biology (HKI), Jena, Germany; 4 Institute for Microbiology, Friedrich Schiller University, Jena, Germany; McGill University, CANADA

## Abstract

The transcription factor Flo8/Som1 controls filamentous growth in *Saccharomyces cerevisiae* and virulence in the plant pathogen *Magnaporthe oryzae*. Flo8/Som1 includes a characteristic N-terminal LUG/LUH-Flo8-single-stranded DNA binding (LUFS) domain and is activated by the cAMP dependent protein kinase A signaling pathway. Heterologous SomA from *Aspergillus fumigatus* rescued in yeast *flo8* mutant strains several phenotypes including adhesion or flocculation in haploids and pseudohyphal growth in diploids, respectively. *A*. *fumigatus* SomA acts similarly to yeast Flo8 on the promoter of *FLO11* fused with reporter gene (*LacZ*) in *S*. *cerevisiae*. *FLO11* expression in yeast requires an activator complex including Flo8 and Mfg1. Furthermore, SomA physically interacts with PtaB, which is related to yeast Mfg1. Loss of the *somA* gene in *A*. *fumigatus* resulted in a slow growth phenotype and a block in asexual development. Only aerial hyphae without further differentiation could be formed. The deletion phenotype was verified by a conditional expression of *somA* using the inducible Tet-on system. A adherence assay with the conditional *somA* expression strain indicated that SomA is required for biofilm formation. A *ptaB* deletion strain showed a similar phenotype supporting that the SomA/PtaB complex controls *A*. *fumigatus* biofilm formation. Transcriptional analysis showed that SomA regulates expression of genes for several transcription factors which control conidiation or adhesion of *A*. *fumigatus*. Infection assays with fertilized chicken eggs as well as with mice revealed that SomA is required for pathogenicity. These data corroborate a complex control function of SomA acting as a central factor of the transcriptional network, which connects adhesion, spore formation and virulence in the opportunistic human pathogen *A*. *fumigatus*.

## Author Summary

Invasive fungal infections affecting immunocompromised patients are emerging worldwide. Among various human fungal pathogens, *Aspergillus fumigatus* is one of the most common molds causing severe invasive aspergillosis in immunocompromised patients. The conidia, which can evade from innate immunity and adhere to epithelial cells of alveoli in human lungs will start to germinate and cause the disease. Currently, the understanding of the molecular mechanisms of adherence of fungal cells to hosts is scarce. The transcription factor Flo8 controls adhesion to biotic or abiotic surfaces and morphological development in baker’s yeast. Flo8 homologues in the dimorphic human pathogenic yeast *Candida albicans* or the filamentous plant pathogen *Magnaporthe oryzae* are required for development and virulence. We found in this study that the Flo8 homologue SomA of *A*. *fumigatus* is required for adhesion and conidiation. Two independent invasive aspergillosis assays using chicken eggs or mouse demonstrated that deletion of the corresponding gene resulted in attenuated virulence. SomA represents an important fungal transcription factor at the interface between adherence, asexual spore formation and pathogenicity in an important opportunistic human pathogen.

## Introduction

Adherence to host cells represents a key step for pathogenesis of bacterial or fungal microorganisms. Prerequisites at the molecular level include cell wall adhesive or hydrophobic proteins, carbohydrate components of the cell wall and the extracellular matrix. Gene families responsible for adherence comprise the *FLO* adhesins (flocculins) of *Saccharomyces cerevisiae* or the *ALS* agglutinins (agglutinins like sequence) of *Candida albicans* [1, 2]. Conidial adherence of the opportunistic human pathogen *Aspergillus fumigatus* requires the hydrophobin RodA, the laminin-binding protein AspF2 or glycans as important constituents of the cell wall [3–5].

Adherence is triggered by different environmental stimuli which are sensed by receptors and which induce various signaling pathways [2, 6]. A prominent example is the cyclic adenosine monophosphate (cAMP) dependent signaling pathway, which is highly conserved from bacteria to mammals. In eukaryotic cells, the cAMP dependent protein kinase A (PKA) signaling pathway is activated by the G protein-coupled receptors [7]. The corresponding Gα subunit activates adenylate cyclases, which convert ATP to cAMP. This secondary messenger binds to the regulatory subunits of PKA. The catalytic subunits of the enzyme are released and activate downstream transcription factors by phosphorylation [7]. The cAMP/PKA pathway plays a crucial role in development and pathogenesis in animal or plant pathogenic fungi such as *C*. *albicans*, *Cryptococcus neoformans*, *Magnaporthe oryzae* and *Ustilago maydis* [8–12]. This link between development, virulence and the cAMP/PKA pathway is conserved in the filamentous fungus and opportunistic pathogen *A*. *fumigatus*. Components of the *A*. *fumigatus* cAMP/PKA pathway include the GpaA and GpaB Gα subunits of the heterotrimertic G protein, the AcyA adenylate cyclase, the PkaR regulatory and the PkaC1/PkaC2 catalytic subunits of PKA. Deletion of *gpaB* or *acyA* results in reduced conidiation and a reduced growth rate in the Δ*acyA* strain [13]. The regulatory PkaR and the catalytic PkaC1 proteins are required to promote germination, growth and accurate conidiation [14–16]. Null mutants of the previous described genes showed attenuated virulence and indicate a role of the cAMP/PKA pathway in the pathogenicity process. This process needs to be further elucidated because of the importance of the *A*. *fumigatus* opportunistic pathogen, which can cause invasive aspergillosis in immunocompromised individuals with mortality rates of more than 60% [17–19].

The cAMP/PKA pathway activates several downstream factors like the *S*. *cerevisiae* transcription factor Flo8, which controls adhesive and filamentous growth. It induces the expression of the *FLO11* gene for an adhesin, which is required for flocculation or the establishment of biofilms in haploid and pseudohyphae formation in diploid yeast strains [2]. The Flo8 counterpart of the dimorphic yeast *C*. *albicans* regulates hyphal development and virulence factors [20]. In both yeasts, Flo8 interacts with additional co-activators as for example Mfg1, which is also required for invasive and hyphal growth [21, 22]. The current knowledge about the transcription factors and their corresponding genes which represent the homologues of the *FLO8* gene are limited. Among filamentous fungi, only the gene for MoSom1 corresponding to Flo8 in the plant pathogenic filamentous fungus *M*. *oryzae* has been examined. The Flo8 counterpart of the dimorphic yeast *C*. *albicans* is the only analyzed protein in a human pathogen. Representatives of constitutively filamentous fungi of human pathogens have not yet been studied. MoSom1 carries like the corresponding yeast protein the N-terminal LUFS (LUG/LUH-Flo8-single-stranded DNA binding) domain and can complement adhesive growth in a Δ*flo8* yeast mutant strain. MoSom1 controls the gene for the hydrophobin MoMpg1, which is required for fungal attachment to plant leaves during infection. Deletion of the *Mosom1* gene results in loss of asexual or sexual development and impairs pathogenicity [23].

The development of *Aspergilli* has been primarily analyzed in the model fungus *Aspergillus nidulans* [24, 25]. The C_2_H_2_ zinc finger transcription factor BrlA represents a central regulator of asexual development in *A*. *nidulans* as well as in *A*. *fumigatus* and controls the formation of vesicles, which are required for conidiation at the top of aerial hyphae. BrlA induces the expression of the downstream *abaA* and *wetA* regulatory genes, which induce differentiation of phialides as spore forming cells and the subsequent maturation of conidia, which represent the asexual spores [26]. The *flbB*, *flbC* and *flbD* regulatory genes are genetically located upstream the expression of *brlA* [25]. MedA and the APSES (Asm1, Phd1, Sok2, Efg1, and StuA) protein StuA regulate transcription of the *brlA* gene in *A*. *nidulans* where they are required for metulae cell formation from vesicles followed by phialide cell formation [27]. *A*. *fumigatus* forms only phialides as asexual spore forming cells but does not produce an additional layer of metulae cells. Though, lack of either MedA or StuA also impairs conidiation in *A*. *fumigatus* where their exact molecular function is yet unknown [28, 29]. Additionally, MedA and StuA control adhesion and virulence in *A*. *fumigatus* by regulating the gene *uge3* encoding uridine diphosphate (UDP)-glucose-epimerase, which is essential for adherence through mediating the synthesis of galactosaminogalactan [30].

The main objective of this study was to examine the function of *A*. *fumigatus* SomA and putative interaction partners. SomA corresponds to the Flo8/Som1 regulator described in other fungi. Our data show that SomA in collaboration with its co-regulator PtaB plays a key role in a transcriptional network controlling conidiation and adhesion and that SomA is required for virulence of filamentous pathogens *A*. *fumigatus*.

## Results

### SomA of *A*. *fumigatus* complements the defects of *S*. *cerevisiae flo8* strains in haploid adhesive and diploid pseudohyphal growth

**Fig 1:**
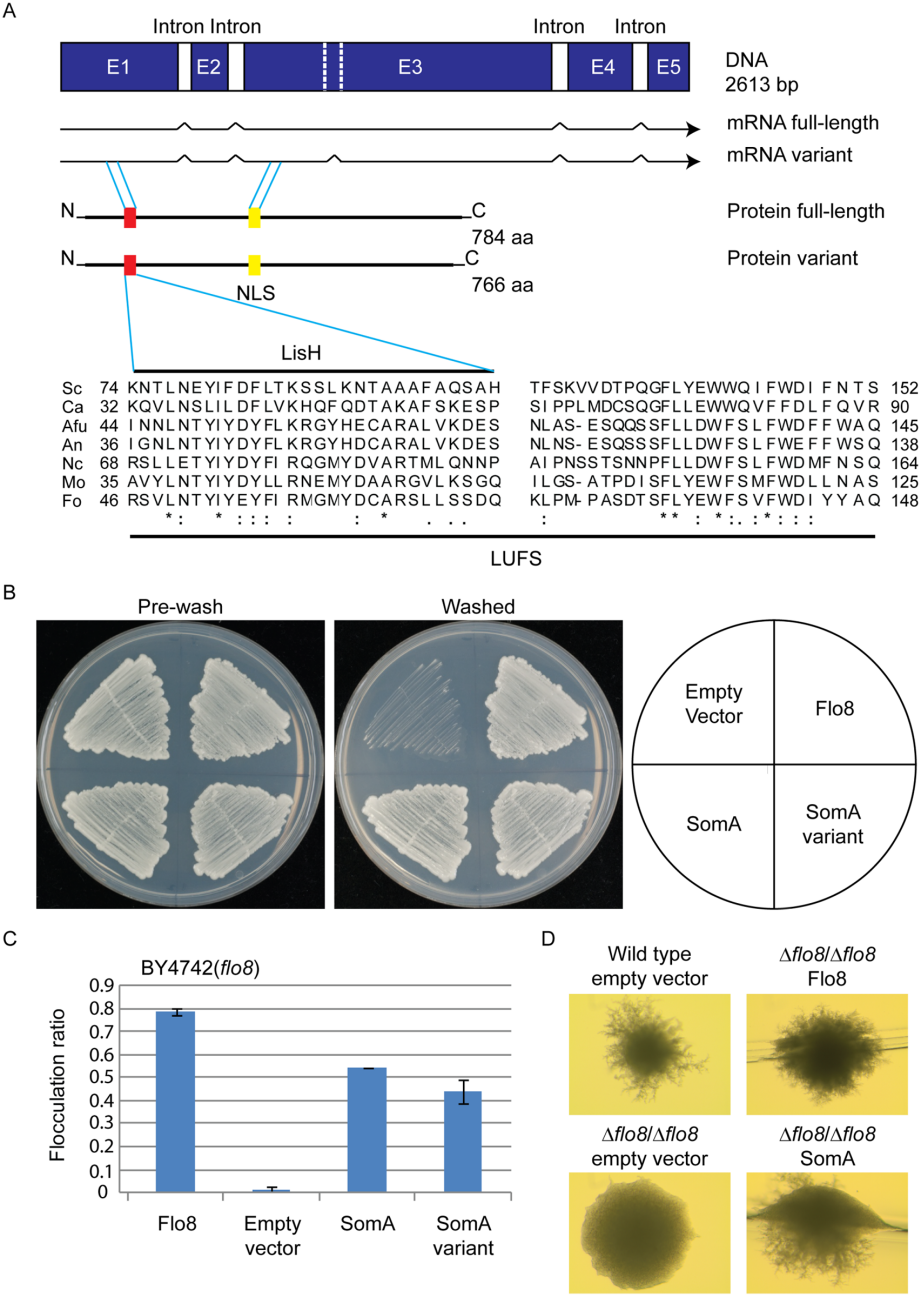
The SomA corresponds to Flo8 and complements developmental phenotypes in *S*. *cerevisiae flo8* mutant strains. (A) The *somA* gene locus results in two mRNA splice variants and two deduced proteins including the LisH motif (red) and a predicted nuclear localization signal (NLS, yellow). The sequence alignments of the LUFS domain including the LisH motif of *A*. *fumigatus* SomA and the corresponding proteins of other fungi. Asterisks indicate identical residues; highly (colon) or modestly (period) similar residues are marked. Abbreviation: Sc, *S*. *cerevisiae*; Ca, *C*. *albicans*; Afu, *A*. *fumigatus*; An, *A*. *nidulans*; Nc, *Neurospora crassa*; Mo, *M*. *oryzae*; Fo, *F*. *oxysporum*. (B) Haploid invasive growth of *S*. *cerevisiae* strain BY4742 (*flo8*) expressing the indicated proteins or empty vector as negative control. Strains were grown on SC-Ura plates for 3 days at 30°C, and the plates were photographed before and after washing under water stream. (C) Flocculation of yeast strain BY4742 (*flo8*) expressing the indicated proteins or empty vector as negative control. Strains were grown in 10 mL SC-Ura medium for one day at 30°C. Graph indicates mean ± standard error of triplicate measurements. (D) Diploid pseudohyphal growth of yeast strain RH2660 (Δ*flo8/*Δ*flo8*) expressing the indicated proteins or empty vector as negative control. The wild type (RH2656) carrying empty vector was used as positive control. Strains were grown on SLAD for 6 days at 30°C and photographed.

Flo8 is a regulator of *S*. *cerevisiae* dimorphism and its counterpart Som1 in the filamentous fungus *M*. *oryzae* is required for plant pathogenicity in rice [20, 23, 31]. These proteins share with the *A*. *fumigatus* protein SomA (AFUA_7G02260) the LUFS domain, which contains a LisH (Lis homology) motif for protein dimerization and tetramerization at the N-terminus (Fig 1A). SomA shows identities of 15.7% and 20.5% to the Flo8 proteins of *S*. *cerevisiae* and *C*. *albicans*, respectively, and 39% to Som1 of *M*. *oryzae*. In addition, there is a conserved nuclear localization signal (NLS) PSPSKRPRLE in filamentous fungi. These data suggest that the proteins derived from all homologous genes have a nuclear function. Exons of *somA* were identified by comparing the DNA sequence of the genomic locus with cDNAs, which were amplified from total mRNA. Sequencing of the resulting plasmid revealed that *somA* carries five exons of a size of 486 bp, 152 bp, 1279 bp, 267 bp and 171 bp (pME4192) resulting in a deduced protein of 784 amino acids with a molecular weight of 84.59 kDa. An additional splice variant (pME4193) was found (Fig 1A) Both splice variants of *somA* could be identified in the Δ*akuA* strain after 20 h vegetative growth in liquid minimal medium (MM).

Cross-species complementation of the *somA* gene was performed in yeast *flo8* mutant strains to verify whether both variants share similar functions with Flo8. Expression of either *somA* or its splice variant (pME4194 and pME4195) under the *MET25* yeast promoter could rescue invasive growth (cell-surface adhesion) in the *flo8* (truncated *FLO8*) haploid mutant (BY4742) on solid agar (Fig 1B). Flocculation (cell-cell adhesion) in liquid medium was complemented similar to Flo8 (pME4197) (Fig 1C). In addition, expression of *somA* in Δ*flo8* diploid strain (RH2660) restored pseudohyphal growth (Fig 1D). These data support that SomA and Flo8 can fulfill similar cellular functions in yeast.

### SomA and Flo8 act through similar promoter sites on *FLO11* expression

**Fig 2:**
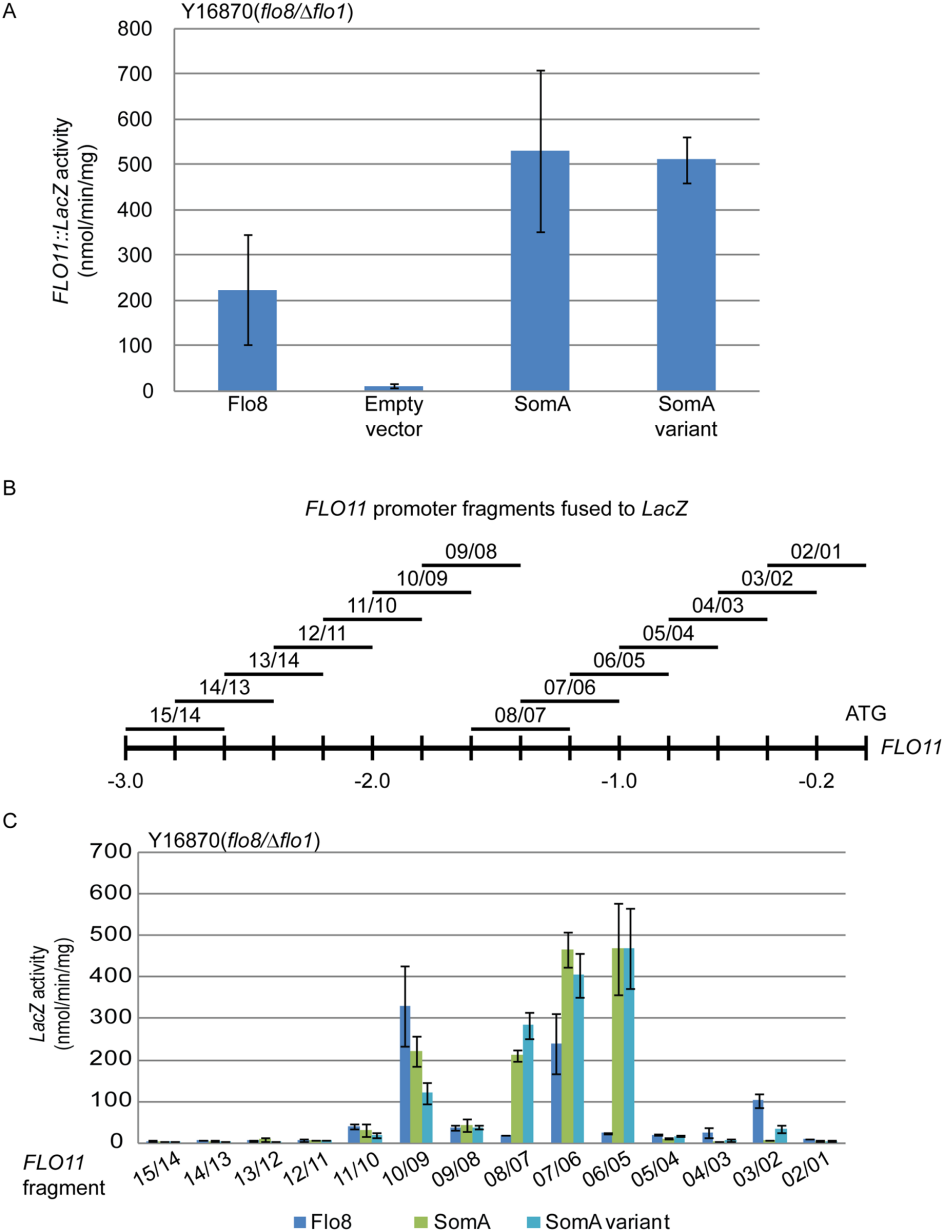
SomA and Flo8 activate *FLO11* expression and act through similar regions of the *FLO11* promoter. (A) Expression of *FLO11*::*LacZ* was determined in haploid Y16870 strain expressing the indicated proteins or empty vector as negative control. (B) Schematic overview of 14 different 400 bp constructs of the *FLO11* promoter region fused to *CYC1*::*LacZ* reporter [31]. (C) Expression of *LacZ* gene fused to different *FLO11* promoter fragments in Y16870 strain expressing the indicated proteins. In all experiments, strains were grown on 10 mL SC-Ura-Leu medium as pre-culture, then 1 mL of samples were inoculated into SC-Ura-Leu-Met medium as main culture for 6 h before the β-galactosidase activities were determined. Graph indicates mean ± standard error of triplicate measurements.

Flo8 is a transcription factor, which binds and promotes transcription of the *FLO11* gene encoding the flocculin Flo11 [32, 33], which is a key determinant for adhesion in yeast [2]. We performed β-galactosidase assays with the 3 kb *FLO11* promoter fused to the bacterial *LacZ* reporter gene to examine whether SomA complements the adhesive phenotypes in *flo8* or Δ*flo8* yeasts (Fig 1B and 1D) by activating *FLO11* gene expression. As shown in Fig 2A, both SomA and its splice variant showed significantly increased *FLO11* promoter driven *LacZ* activity in comparison to the mutant strain transformed with the empty plasmid.

We took a more detailed look at the *FLO11* promoter to determine whether SomA and Flo8 bind to similar regions of the promoter. A set of 14 reporter constructs containing 400 bp *FLO11* promoter fragments that overlap by 200 bp [34] (Fig 2B) was analyzed in the *flo8/*Δ*flo1* yeast strain (Y16870). As shown in Fig 2C, two promoter regions were affected by both Flo8 and SomA. Comparison of Fig 2B and 2C indicated that these two regions are located at 1.8 kb and 1.2 kb upstream of the start codon of *FLO11*. SomA presumably recognizes two additional regions located at 1.4 kb and 1 kb upstream of the *FLO11* open reading frame. These data indicate that SomA and Flo8 share molecular functions in recognizing and controlling similar regions of the *FLO11* promoter and hence complemented adhesion and filamentous growth in *flo8* and Δ*flo8* yeast strains.

### SomA physically interacts with PtaB

**Fig 3:**
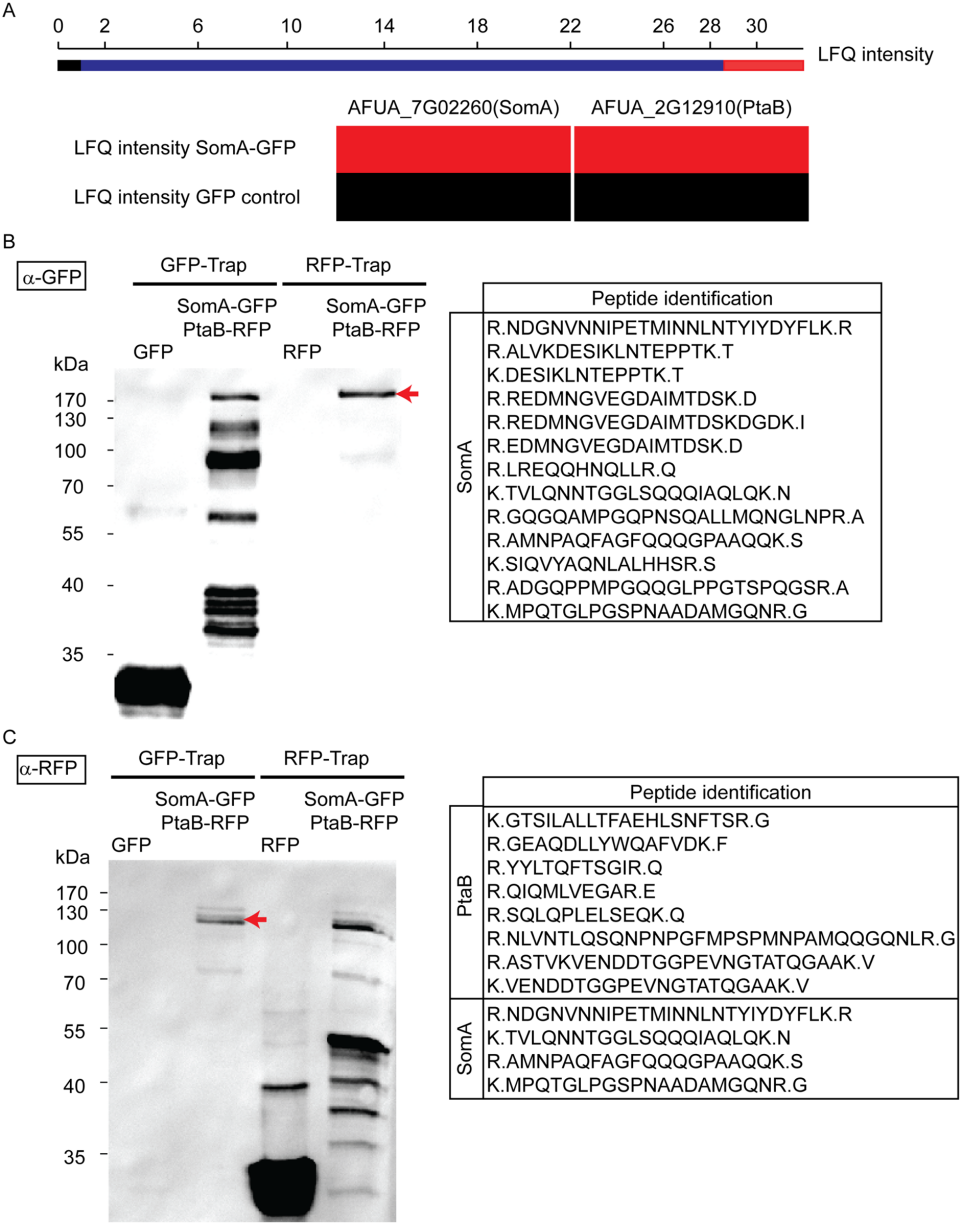
SomA interacts with PtaB in *A*. *fumigatus*. (A) The abundance of SomA and PtaB was measured by LC/MS and estimated based on MaxQuant’s logarithmized label free quantification (log2 LFQ) intensities. High and intermediate LFQ intensities are shown in red and blue. Absence of peptides and low LFQ intensities are presented in black. (B) Western hybridization of GFP-Trap and RFP-Trap enrichments with α-GFP antibody. The single band in the RFP-Trap indicated by an arrow was identified as SomA-GFP by LC/MS with the given peptides. (C) Reciprocal western to (B) but an α-RFP antibody instead of the α-GFP antibody as a different probe. The double band (arrow) correspond to PtaB and SomA by LC/MS. For all experiments, the strains were grown in MM medium for 24 h at 37°C. Protein extracts were performed with either GFP-Trap or RFP-Trap beads and the eluted proteins were separated by 12% SDS-PAGE.

Yeast Flo8 is part of a protein complex required for regulating cellular development, and Mfg1 represents another subunit of this complex [21, 22]. We analyzed whether the similarity of SomA to Flo8 and the similar function of both proteins are reflected by similar interaction partners of SomA in *A*. *fumigatus*. A GFP tagged *somA* gene was constructed (AfGB75) to identify interaction partners of SomA. A GFP-Trap was performed and the recruited proteins were analyzed by LC/MS. Proteins identified in the GFP control strain were considered as unspecific background identifications (LC/MS raw data in S1 Table). Apart from SomA itself, the PtaB protein (AFUA_2G12910), a homologue of yeast Mfg1, was identified by LC/MS. This protein was absent in GFP control strain (Fig 3A). The detailed LC/MS data are shown in S2 Table.

We further performed a co-immunoprecipitation to verify whether PtaB and SomA are interaction partners (Fig 3B and 3C). A strain expressing SomA-GFP and PtaB-RFP fusion proteins was constructed (AfGB117). Application of the α-GFP antibody recognized GFP in the trap enrichment of the GFP control strain (Fig 3B). Several signals in the GFP-Trap of the strain expressing both fusions (*somA-gfp/ptaB-rfp*) presumably represent SomA-GFP and its degradation products. Only a single signal in the RFP-Trap was detected by the α-GFP antibody. The single signal (arrow, Fig 3B) at approximately 170 kDa was verified by LC/MS as SomA-GFP. This suggests that the SomA-GFP protein has been recruited through the RFP-Trap by PtaB-RFP. The reciprocal experiment using an α-RFP antibody and the same trap enrichments resulted in the recognition of RFP in the RFP control strain and several signals in the RFP-Trap presumably representing PtaB-RFP and its derivatives. Two signals at 120 kDa in the GFP trap enrichment (arrow, Fig 3C) were identified by the α-RFP antibody and were determined as the PtaB-RFP protein with a calculated size of 106 kDa by LC/MS. In addition, several SomA-GFP peptides were identified by LC/MS which presumably correspond to the SomA-GFP degradation products which are visible in Fig 3B (GFP-Trap lane). Taken together, these data suggest that SomA and PtaB physically interact in *A*. *fumigatus* similar to their counterparts Flo8 and Mfg1 in the two yeasts *S*. *cerevisiae* and *C*. *albicans* [22].

### Deletion of the *somA* gene blocks *A*. *fumigatus* asexual development at aerial hyphae

**Fig 4:**
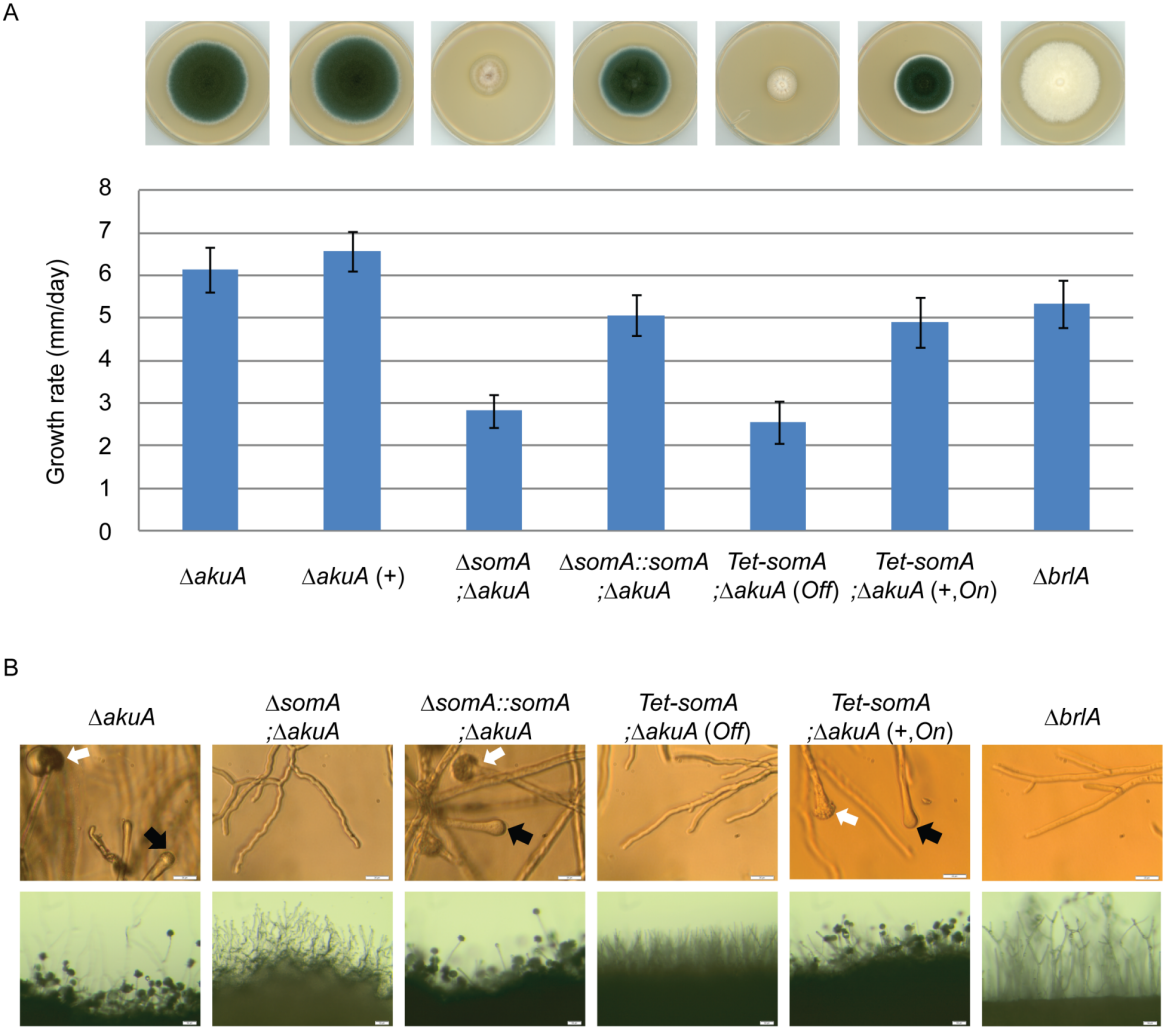
SomA promotes growth and conidia formation of *A*. *fumigatus*. (A) Colony morphology and growth rate of the indicated strains. All strains were grown on either MM plate or MM plate with 5 mg/L doxycycline indicated as (+) for 5 days at 37°C. Values in the graph are indicated as means ± standard error. (B) Morphology of conidiation in the indicated strains. Upper panel: Strains were grown on MM or MM with 5 mg/L doxycycline agar-coated slides for 28 h at 37°C. The open arrows indicate conidiophores and filled arrows represent the vesicles for sporulation. Lower panel: Strains were grown on MM or MM with 5 mg/L doxycycline agar-slides for 28 h at 37°C. Scale bars represent 20 μm (upper panel) and 50 μm (lower panel). Strains grow on MM agar with doxycycline indicated as (+). For all experiments, the *Tet-somA* was induced (*On* state) at the present of doxycycline.

The *somA* gene was deleted in a Δ*akuA* background strain (AfS35) to analyze the function of this gene in correlation with growth, adhesion and development. Cultivation on solid MM plates revealed slow growth (2.8 mm/day) of the Δ*somA* strain (AfGB77) in comparison to Δ*akuA* strain (6.1 mm/day). This Δ*somA* growth phenotype was verified by complementation with the respective wild type gene. The complemented strain (AfGB105) showed improved growth rate (5.1 mm/day), indistinguishable from Δ*akuA* strain (Fig 4A). We also analyzed PtaB as the physical interaction partner by genetic analysis. The deletion of *ptaB* (AfGB115) resulted also in a reduced growth rate phenotype (4.6 mm/day) which was less pronounced in comparison to the Δ*somA* phenotype. In addition, a delayed conidiation was observed in the *ptaB* null mutant (arrow, S1 Fig). The growth defect and the delayed asexual development of the Δ*ptaB* strain were restored by complementation with a *ptaB-rfp* fusion (S1 Fig).

Asexual spores are normally produced at conidiophores consisting of aerial hyphae with a vesicle on top where the conidia are pinched off [27]. The Δ*somA* strain formed exclusively aerial hyphae and was incapable of forming conidiophores. To have a detailed look on conidiation, the strains were inoculated on MM-agar coated microscope slides or MM agar on microscope slides and incubated for 28 h at 37°C. As shown in Fig 4B, the Δ*somA* mutant showed no mature conidiophores. In contrast, the Δ*akuA* strain and the *somA* complemented strain revealed conidiophores (white arrow) and vesicle formation (black arrow) on top of the aerial hyphae. Furthermore, macroscopic inspection indicated that the Δ*akuA* strain produces aerial hyphae and conidiophores similar to the *somA* complemented strain. In contrast, the Δ*somA* mutant showed only aerial hyphae (Fig 4B). The defect in vesicle formation of the Δ*somA* mutant was similar to the defect in a Δ*brlA* strain except of the growth retardation (Fig 4A) [35]. We analyzed the SomA dependent step in asexual development in more detail. A *Tet-somA* strain (AfGB74) was constructed by replacing the promoter region with the inducible Tet-On system [36] which could conditionally express the *somA* gene upon addition of doxycycline to the medium.

The growth and conidiation phenotype of the Δ*akuA* strain were not affected by the presence of doxycycline (Fig 4A). The *Tet-somA* strain grew as slowly as the Δ*somA* mutant and had severely impaired sporulation when doxycycline was absent (*Off* state). In contrast, these impaired phenotypes were complemented when the promoter was induced by doxycycline (Fig 4A). Further observation showed that the *Tet-somA* strain revealed conidiophores (white arrow) and vesicle formation (black arrow) on top of the aerial hyphae as the Δ*akuA* strain only under inducing conditions (Fig 4B). Taken together, these results support a function of SomA in asexual development and fungal growth.

### SomA and PtaB are required for biofilm formation

**Fig 5:**
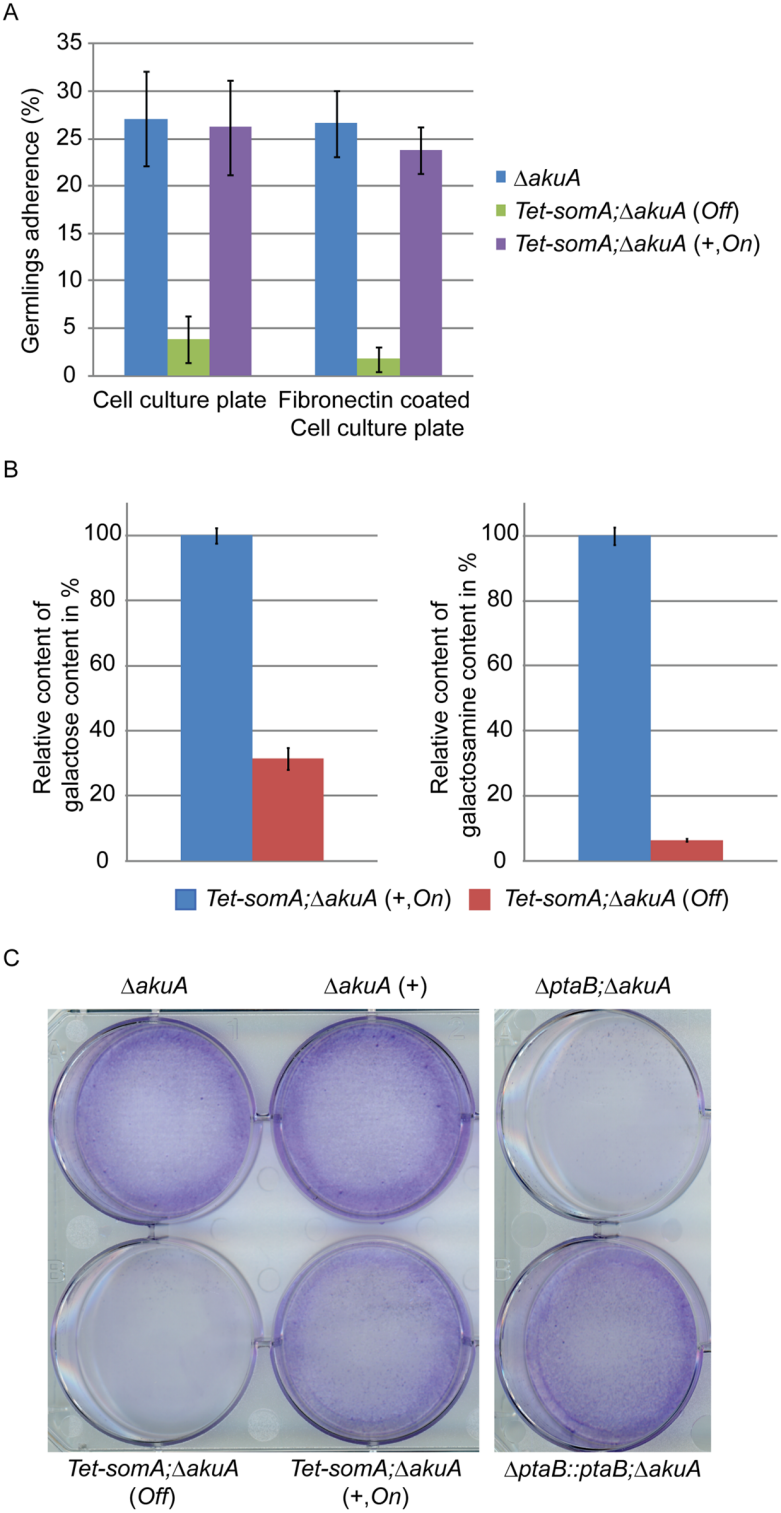
SomA and PtaB are involved in biofilm formation in *A*. *fumigatus*. (A) Germlings adherence of the indicated strains to plastic surfaces and fibronectin coated wells. Addition of 5 mg/L doxycycline is indicated with (+). Values in the graph are indicated as means ± standard error with triplicate determinations. (B) Galactosaminogalactan (GAG) assay for the *Tet-somA* strain. Results amounts of galactose and galactosamine are shown. Levels for the *Tet-somA On* state were set to 100%. (C) Biofilm formation of the indicated strains inoculated on polystyrene plates for 24 h. The wells were washed with PBS and stained with crystal violet. Addition of 5 mg/L doxycycline is indicated with (+). For all experiments, the *Tet-somA* was induced (*On* state) at the present of doxycycline.

Flo8 is required for adherence of *S*. *cerevisiae* by regulating *FLO* gene expression [2]. Therefore, the impact of the loss of the *somA* gene on the adherence to plastic or fibronectin were examined. Due to the fact that the Δ*somA* mutant has a defect in asexual development, we used the *Tet-somA* strain to perform the adherence assay and the Δ*akuA* strain was used as control. As a pilot test the adherence of germlings was tested. Germlings of the Δ*akuA* strain and *Tet-somA* mutant (*On* state) displayed 25% adherence to polystyrene plates and fibronectin-coated plates. In contrast, the *Tet-somA* germlings (*Off* state) showed only 5% adherence to both surfaces (Fig 5A).

The polysaccharide galactosaminogalactan (GAG) from the fungal cell wall is composed of α1,4-linked galactose and N-acetylgalactosamine and plays a role in fungal adherence [30]. We tested whether the loss of germling adhesion in the *Tet-somA* mutant (*Off* state) is due to reduced GAG production. We cultivated the strain under inducing and non-inducing conditions and after precipitation and hydrolysis of GAG, the amounts of galactose and galactosamine were measured by GC-MS [30]. The amount of galactose was reduced to 31% and the amount of galactosamine was reduced to 6% in the *Tet-somA* strain (*Off*) compared to the *Tet-somA* strain (*On*), respectively (Fig 5B).

The yeast Flo8-Mfg1 complex is required for biofilm formation [22]. We analyzed whether SomA and PtaB play a similar role in the *A*. *fumigatus* life style. As shown in Fig 5C, the hyphae of the *Tet*-*somA* strain (*On*) formed biofilm when the promoter was induced by doxycycline (+). The Δ*akuA* strain with (+) or without the drug showed similar biofilm formation (Fig 5C). In contrast, the complete mycelium was washed off when the *Tet*-*somA* strain was at *Off* state. A similar phenotype was detected for PtaB. The Δ*ptaB* mutant strain resulted in a defect of biofilm formation and this phenotype could be rescued by re-introducing the *ptaB-rfp* fused gene in the deletion strain (Fig 5C).

Taken together, these data show a common function of SomA and PtaB in biofilm formation. Furthermore, SomA is required for germling adherence to plastic surfaces or fibronectin and GAG production.

### SomA controls the expression of genes related to the process of conidiation and adherence in *A*. *fumigatus*

**Fig 6:**
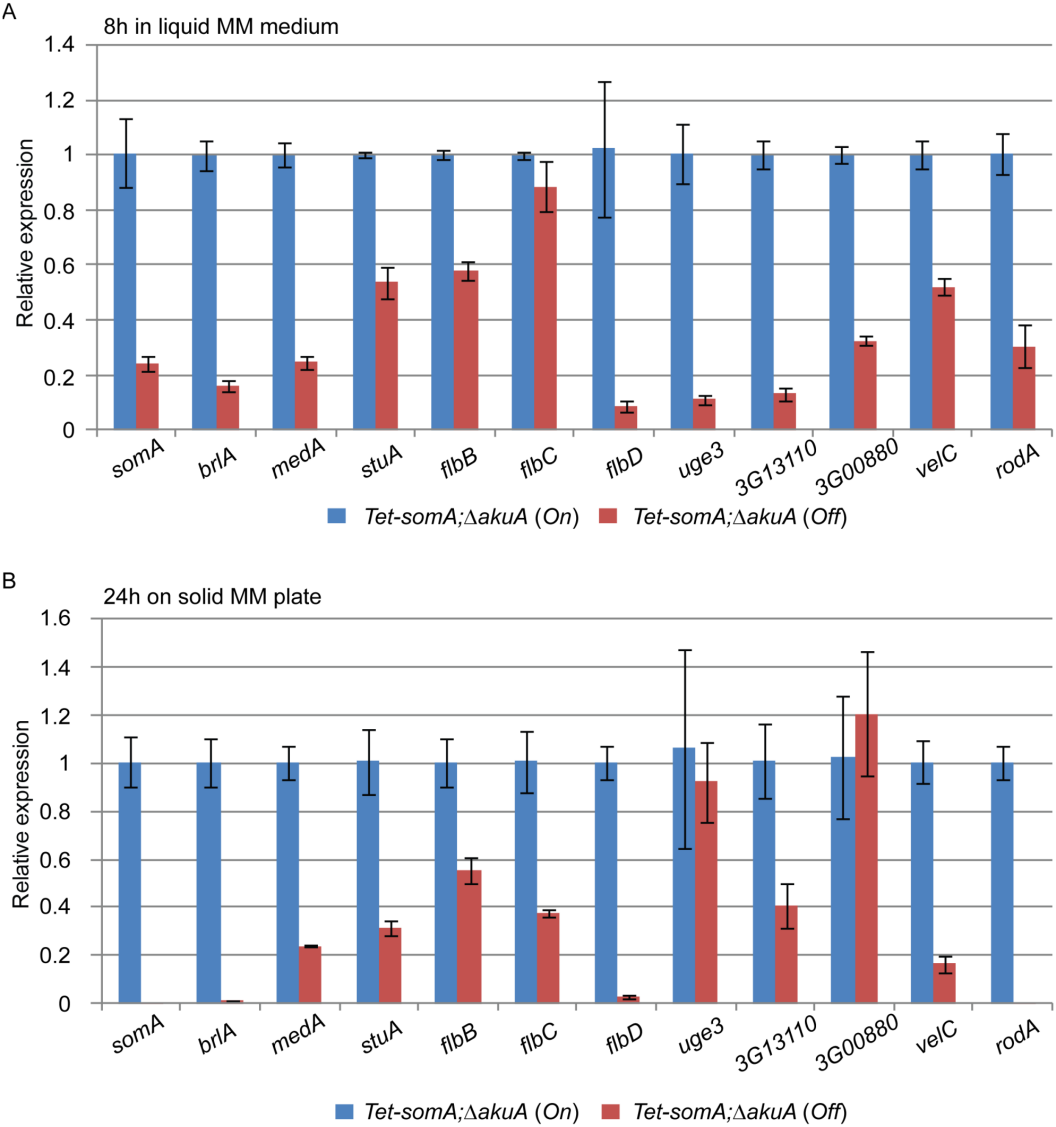
SomA regulates genes for conidiation and adhesion. Relative expression of genes encoding proteins that regulate conidiation and adhesion in the *Tet-somA* strain. The *Tet-somA* strain was cultivated in liquid MM medium for 18 h at 37°C and shifted to (A) liquid MM medium for 8 h at 37°C and (B) solid MM plate for 24 h at 37°C. Addition of 5 mg/L doxycycline is indicated as (*On*). Levels for the *Tet-somA On* state were set to 1. Graph indicates mean ± standard errors from two independent experiments.

The cellular function of SomA as a transcription factor involved in asexual development and adherence was examined by quantitative transcript analysis of putative target genes. We could show that the *Tet-somA* strain has a similar asexual development as the Δ*somA* mutant when doxycycline is absent (Fig 4). In addition, we showed that SomA plays an important role in adherence and GAG production using the *Tet-somA* strain (Fig 5). Hence, we used the *Tet-somA* strain to test the role of SomA in gene regulation. The Δ*akuA* mutant and the *Tet-somA* strain were incubated in liquid minimal medium (MM) for 18 h. Afterwards, the mycelium was shifted to liquid MM for 8 h and solid MM plate for 24 h with or without doxycycline. The drug had no effect on gene expressions in the Δ*akuA* mutant (S2 Fig). Transcript analysis revealed that the *Tet-somA* strain (*Off*) abolished *brlA* expression in contrast to the *On* state of the *Tet-somA* strain (Fig 6). In *A*. *fumigatus*, FlbB is necessary for *flbD* expression and FlbD might be essential for expression of *brlA* [25]. The expression of *flbBCD* genes in the *Tet-somA* strain was decreased in the *Off* state in comparison to the *On* state (Fig 6). The velvet domain protein family and LaeA also control fungal development and secondary metabolism in filamentous fungi including conidiation [24, 37, 38]. The expression of members of the velvet domain family was not significantly affected except for transcription of the *velC* gene, which was impaired by the *Tet-somA Off* state (Fig 6). These results suggest a broader role of SomA in fungal development.

Gravelat *et al* (2013) showed that *medA* and *stuA* genes are required for adhesion and regulate some putative adhesins [28, 30] and the transcript levels of *medA* and *stuA* were significantly reduced in the *Tet-somA Off* state (Fig 6). Possible adherence genes located downstream of the *medA* and *stuA* genes were further evaluated. Three genes (AFUA_3G13110, AFUA_3G00880 and *uge3*) encoding possible adherence-associated proteins with high scores in bioinformatic prediction [39] were analyzed. The transcript levels of all three genes are reduced in the absence of *somA* (*Off* state) (Fig 6). A similar transcript analysis was also observed in the Δ*somA* mutant in comparison to the Δ*akuA* background strain and the *somA* complemented strain (S3 Fig).

Deletion of the SomA interaction partner PtaB also resulted in a delayed conidiation (S1 Fig) and, a defect of biofilm formation (Fig 5C). This suggests that PtaB might also contribute to the SomA control of gene transcriptions. The transcript levels showed that the Δ*ptaB* mutant strain had a significant effect on the expression of the development and adherence related genes which are also controlled by SomA (S4 Fig).

**Fig 7:**
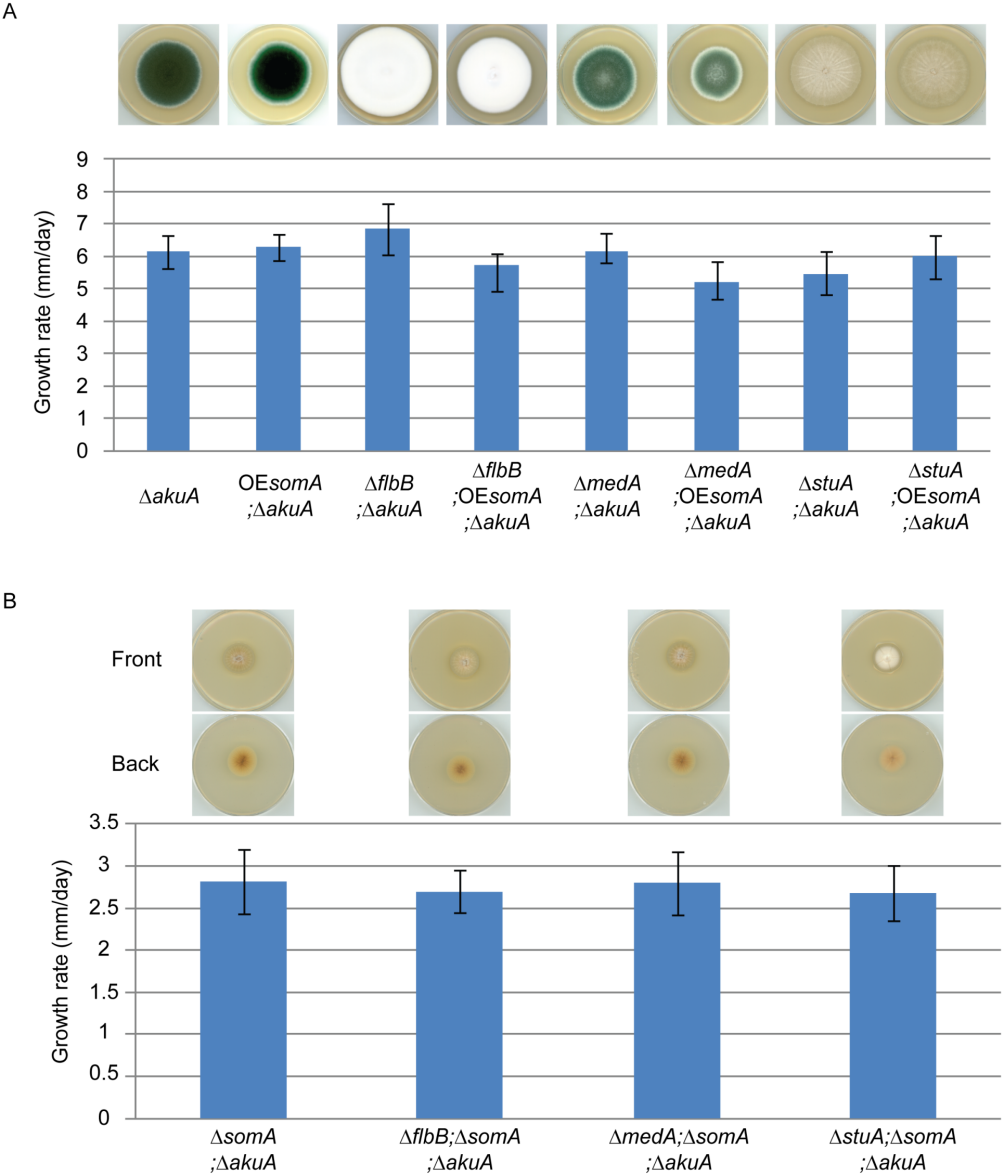
SomA acts upstream of *flbB*, *medA* and *stuA* genes. (A) Colony morphology and growth rate of the indicated strains. (B) Colony morphology and growth rate of the corresponding strains. All strains were grown on MM plate for 5 days at 37°C. Values in the graph are indicated as means ± standard error.

SomA, FlbB, MedA and StuA represent fungal transcription factors controlling a complex developmental regulatory transcriptional network. To identify the interaction between SomA and these three transcription factors, an epistatic analysis was performed. The overexpression of *somA* did not change the phenotype of either Δ*akuA* background, Δ*flbB*, Δ*medA* or Δ*stuA* mutant strains and had also no significant effect on colony growth (Fig 7A). Double deletion strains revealed a different picture. An additional *somA* deletion in the Δ*flbB* background resulted no more in a Δ*flbB* but in a Δ*somA* colony phenotype including the reduced growth rate of the colony. The same phenotype indistinguishable from the Δ*somA* mutant was observed in the double mutant strain with Δ*medA* (Fig 7B). The Δ*stuA* Δ*somA* double mutant (AfGB114) showed a more complex phenotype which does not completely correspond to the Δ*somA* deletion. More aerial hyphae on the surface and a lighter color on the back are visible compared to Δ*somA* single or the other double deletion strains.

Taken together, our combined genetic and transcriptional analysis supports that SomA regulates asexual development regulatory genes *flbB* and *flbD* and, through this pathway, affects the *brlA* master gene of conidiation. There is presumably an additional combinatory effect between SomA and the regulator StuA. The network of SomA, PtaB, StuA and MedA finally results in a SomA-mediated control of various adhesins encoding genes in *A*. *fumigatus*.

### SomA is required for virulence in an egg and a mouse infection model

SomA is presumably required for adhesion by affecting *medA* expression and a Δ*medA* deletion results in reduced virulence in a mice model [28]. Hence, we addressed whether SomA plays a role in virulence in animals. We established the Tet-On system in an egg infection model as a pilot study to carry out the virulence experiments. This model mimics the pulmonary invasive aspergillosis model in mice by infecting the chorioallantoic membrane in eggs [40].

**Fig 8:**
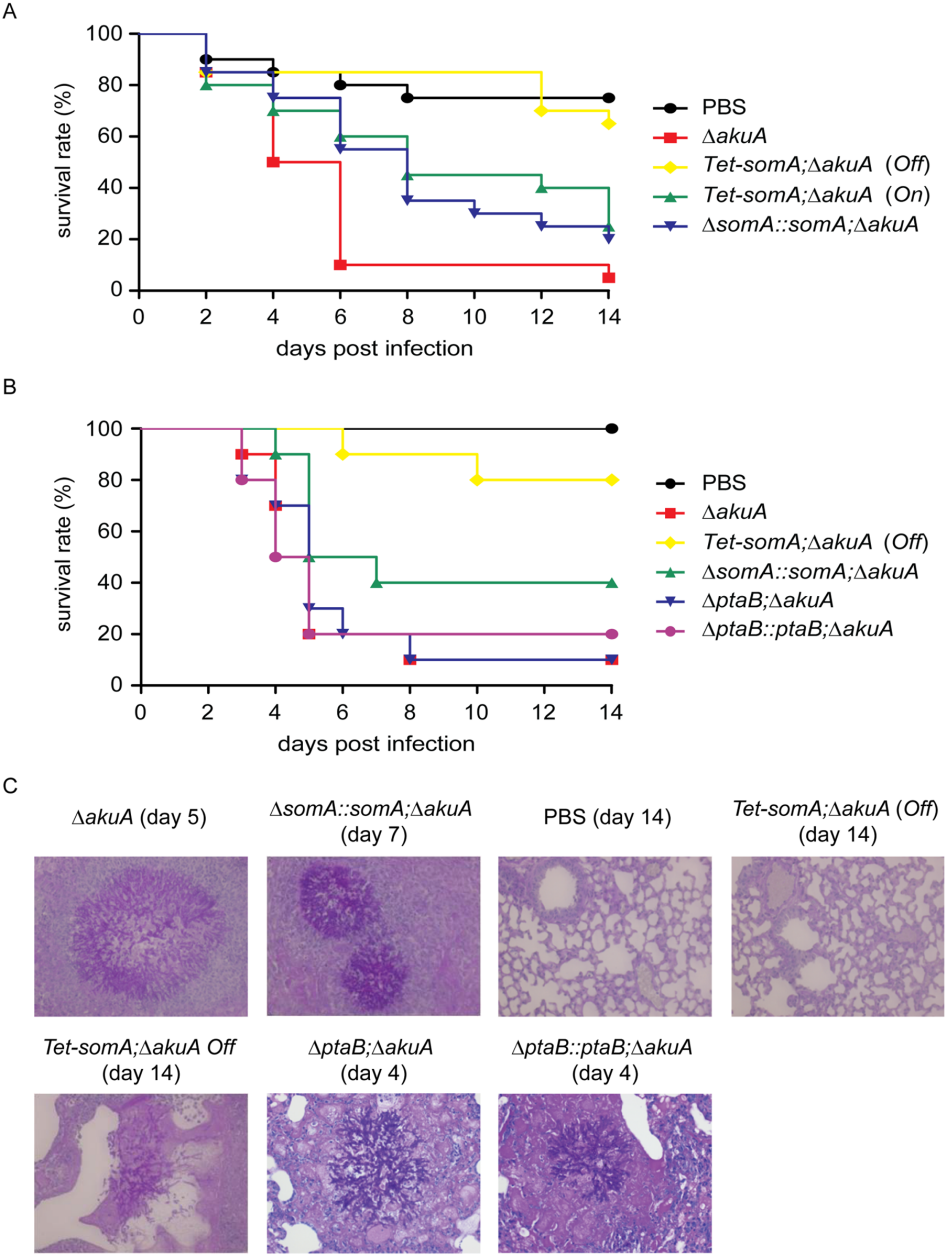
SomA is required for virulence in an egg and a mouse model of invasive aspergillosis. (A) Survival rate of eggs inoculated with the indicated strains. Addition of doxycycline was not performed in the *somA* complemented mutant and *Tet-somA Off* state. (B) Survival rate of mice infected with the indicated strains. (C) Histological pictures of mouse lungs obtained from indicated dates after infection with the corresponding strains or PBS.

In an egg infection model, the Δ*somA* mutant was not included due to the severely impaired conidiation. The eggs infected with the inactive *Tet-somA* strain without doxycycline (*Off*) had no significant difference in mortality of infected eggs compared to the PBS control (p = 0.58; log-rank test). The *Tet-somA* (*Off*) showed attenuated virulence compared to the Δ*akuA* strain, the *somA* complemented and the *Tet-somA* (*On*) (p<0.05). In contrast, the *On* state of the *Tet-somA* strain which was induced by doxycycline showed similar virulence to the Δ*akuA* strain or the *somA* complemented strain (Fig 8A).

We verified the egg infection experiments by virulence assays of the mutant compared to the Δ*akuA* background strain in a mouse infection model for pulmonary aspergillosis [41]. As in the egg model, the Δ*somA* mutant was not included due to the severely impaired conidiation. Addition of doxycycline in mouse model was not performed due to the fact that the *somA* complemented strain and the *Tet-somA* (*On*) strain show the virulence in the egg infection (Fig 8A). In this model, the *Tet-somA* mutant (*Off*) displayed attenuation in virulence, which was statistically significant compared to the Δ*akuA* background and the *somA* complemented strain (p<0.05) (Fig 8B). In neutropenic mice, a cellular immune response is severely restricted and development of invasive aspergillosis is characterized by massive invasive growth of the fungus. Accordingly, the presence of invasive mycelia was confirmed by histopathology in mice infected with the Δ*akuA* background and the complemented conidia. Even the two mice that died after infection with *Tet-somA* (*Off*) showed fungal growth within the lung tissue. However, no mycelium could be found in the majority of mice that survived the infection with *Tet-somA* conidia (*Off*) (Fig 8C). We had shown that the Δ*ptaB* mutant had a defect of biofilm formation (Fig 5C) and PtaB interacts with SomA (Fig 3). This suggests that PtaB might also contribute to the SomA control of pathogenesis. However, a mice infection model showed that the Δ*ptaB* mutant strain had normal virulence and invasive mycelia as the Δ*akuA* background strain (Fig 8B and 8C).

The resistance of oxidative stress and cell wall integrity are important virulent factors in *A*. *fumigatus* infection [42, 43]. Due to the fact that loss of *somA* was avirulent whereas the Δ*ptaB* strain had normal virulence (Fig 8B), we performed a stress test to study the function of these two genes in stress response. As shown in S5 Fig, the *ptaB* null strain was resistant to H_2_O_2_ (3 mM) in comparison to the Δ*akuA* background strain. In contrast to this result the loss of *somA* does not increase resistance to H_2_O_2_.

Taken together, these data show that the Tet-On system is functional in the egg infection assay of *A*. *fumigatus*. The egg as well as the mouse model as established infection assays support that SomA is contributing to virulence of the opportunistic fungal pathogen *A*. *fumigatus*.

## Discussion

The current understanding of Flo8/Som1 homologues and their role in adhesion and virulence is primarily based on yeasts with their dimorphic life style switching between a single cell yeast growth form and a pseudohyphal or hyphal growth mode [20, 31]. In addition, Som1 had been analyzed in plant pathogenic and saprophytic filamentous fungi [23, 44]. Here, we show that similar to the Flo8-Mfg1 complex in yeast the corresponding pair SomA-PtaB is required for biofilm formation in the opportunistic human pathogenic filamentous fungus *A*. *fumigatus*. Application of the Tet-On system revealed that the Flo8/Som1 counterpart SomA of this fungus functions in development and virulence in embryonated hen egg as well as a mouse infection model.

The mechanism of adherence has most extensively been studied in the yeast *S*. *cerevisiae*. Since adhesion is highly correlated with pathogenicity in fungi, *S*. *cerevisiae* has been used as a model for detecting adhesins encoding genes and identifying control genes of adhesion from *C*. *albicans* [45] and the filamentous fungus *Verticillium longisporum* [46]. Flo8 is one of the most prominent yeast regulators of adhesion and it had been demonstrated that Flo8 functions downstream of the cAMP/PKA pathway [47]. The binding of Flo8 to target promoters is regulated in budding yeast by Tpk2, which is one of the catalytic subunits of PKA, and loss of either Flo8 or Tpk2 blocks pseudohyphal growth [48]. We showed that heterologous SomA protein complements the defects of *flo8* yeast in haploid adhesive (cell-cell and cell-surface) and diploid pseudohyphal filamentous growth, which require the expression of *FLO11* or *FLO1* [2, 32]. Furthermore, the expression of *FLO11* could be activated by SomA in *flo8* yeast. These results suggest that SomA might be activated by Tpk2 in *S*. *cerevisiae* and it can activate downstream genes as *FLO11*.

Higher expression levels of *FLO11*::*LacZ* reporter were measured by heterologous SomA compared to the Flo8. Further analysis of *FLO11* promoter regions indicated that SomA binds to two similar promoter regions as Flo8 and two additional regions. All these four regions contain the Flo8 consensus binding sequence TTTGC [49]. The higher promoter binding activity of SomA might be due to a higher overall binding affinity to the *FLO11* promoter compared to Flo8. Flocculation requires both adhesins Flo1 as well as Flo11 [33, 50]. The observed decreased flocculation by SomA suggests a poor binding activity to the promoter of *FLO1*. The fact that heterologous SomA activates the expression of *FLO11* by binding to similar regions of the promoter as Flo8 supports an evolutionary conserved strategy of gene activation in yeast and filamentous fungi.

Flo8/Som1 is a transcription factor that regulates downstream targets together with other interaction partners [22, 23]. Here, we showed that SomA interacts with PtaB, the *A*. *fumigatus* homologue of yeast Mfg1. PtaB and Mfg1 proteins are members of the LIM-domain binding protein family, which play a role in development in eukaryotic cells [51, 52]. In *S*. *cerevisiae*, the Mfg1 protein forms even a ternary complex with Mss11 and Flo8 leading to efficient *FLO11* expression and hence mediating invasive growth and pseudohyphal formation [22]. Deletion of either partner gene leads to the loss of these growth forms. In *C*. *albicans*, this heterotrimeric complex is also required for hyphal growth [21], but overexpression of *FLO8* complemented the defect of Δ*mss11* in hyphal growth [53]. This indicated that Flo8 and Mss11 might share functions in development. The closest relative of an Mss11 encoding gene in *A*. *fumigatus* is *somA* with no other putative paralogue present in the genome. One possible explanation is that the yeast Flo8-Mss11-Mfg1 complex might correspond to a SomA-SomA-PtaB complex in *A*. *fumigatus*. The *MSS11* gene in yeasts might be either a product of gene duplication in yeasts or might have been lost in filamentous fungi as the *Aspergilli*.

Increased protein sizes of SomA-GFP and PtaB-RFP were detected with the respective antibodies in Western experiments (Fig 3B and 3C). These results imply that SomA-GFP and PtaB-RFP is modified post-translationally. Bioinformatic prediction tools [54] suggest 10 putative ubiquitination sites (medium confidence) in SomA and three additional sites in the deduced PtaB primary amino acid sequence. Consistently, SomA-GFP is simultaneously recognized with α-Ubi and α-GFP antibodies (S6 Fig).

Asexual development in *Aspergilli* is a morphological change, which is reminiscent to the dimorphic life style of yeasts. Aerial hyphae are formed which can differentiate into conidiophores, containing many single cell conidia with a single nucleus per cell. These asexual spores are released into the air for dispersal of the fungus [25, 27]. The combined data of the deletion analysis and the regulated promoter suggest that SomA is a regulator of asexual spore formation and is controlling developmental steps after the formation of aerial hyphae during the asexual cycle (Fig 4B). The similar developmental phenotypes in vesicle formation between the Δ*somA* (AfGB77) and the Δ*brlA* (A1176) strain and the fact that SomA regulates the expression of *flbB*, *flbD* and *brlA* genes suggest that SomA and BrlA might be part of the same regulatory pathway. This is consistent with earlier findings where lack of conidiation was also observed in *M*. *oryzae* and *A*. *nidulans* when Som1 is inactivated [23, 44].

The connection between Flo8/Som1 and the PKA pathway seems to be conserved between yeasts and filamentous fungi. The Δ*somA* strain in *A*. *fumigatus* was reduced in its growth rate in comparison to the wild type and resembles the Δ*acyA* mutant phenotype, which is deficient in the adenylate cyclase producing cAMP where growth was reduced and nearly no conidiation was observed [13]. MoSom1 interacts with the CpkA catalytic subunit of protein kinase A [23]. *A*. *fumigatus pkaC1 and pkaC2* encode two cAMP dependent PKA catalytic subunits. PkaC1 belongs to the class I PKAs similar to Tpk proteins of *S*. *cerevisiae* whereas PkaC2 is dispensable for conidiation. In contrast, PkaC1 is responsible for conidiation and vegetative growth [15]. This suggests that the protein kinase PkaC1 may regulate activation of SomA and subsequently control asexual development.

**Fig 9:**
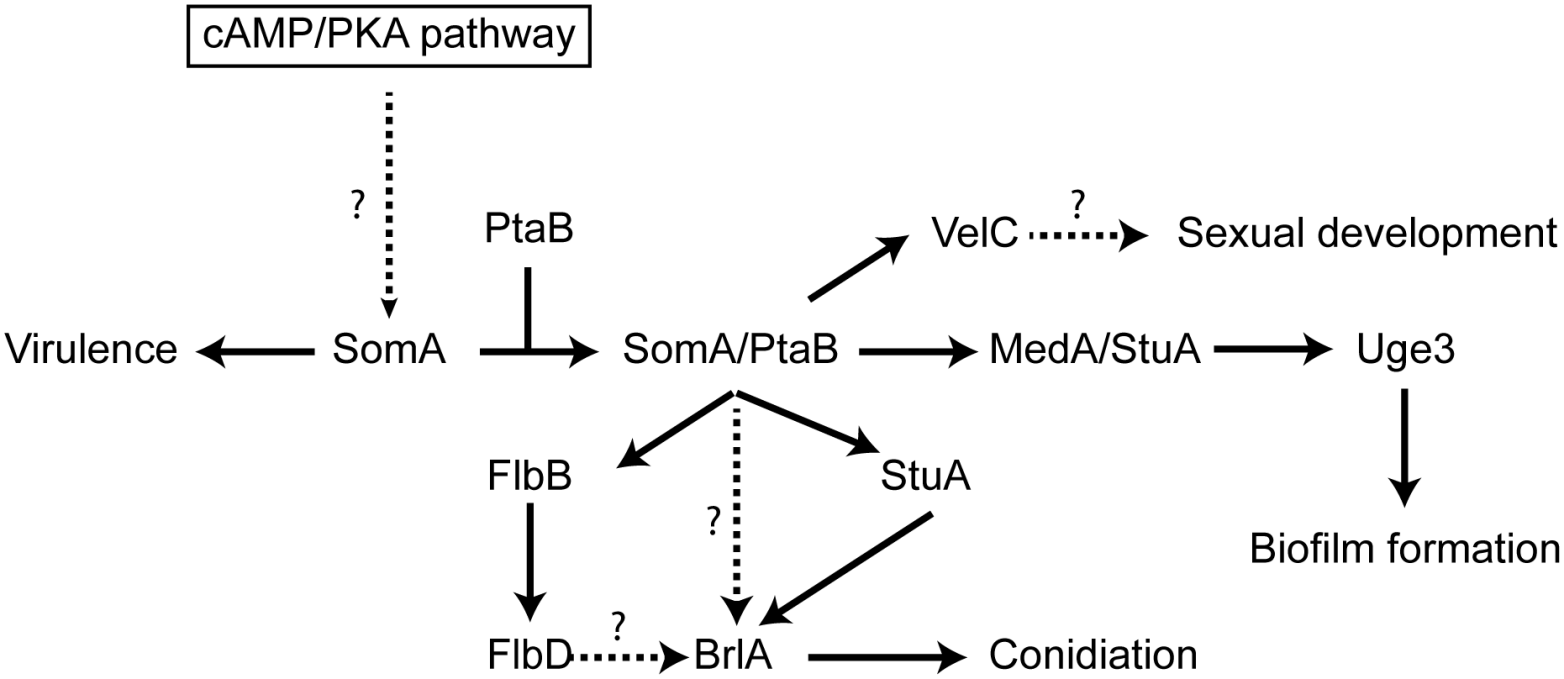
Model of the SomA and SomA-PtaB genetic network in *A*. *fumigatus*. The solid arrows indicate the presented data and the results of previous studies [25, 28–30, 56]. We showed that SomA/PtaB complex is a transcriptional activator for downstream targets (Fig 6 and S3 Fig) and these proteins had different cellular functions in *A*. *fumigatus* (See Discussion).

SomA controls conidiation and adhesion primarily by affecting the expression of the three regulatory genes *flbB*, *stuA* and *medA* (Fig 9). FlbB is a bZIP transcription factor which controls together with the cMyb transcription factor FlbD the expression of the major regulatory gene *brlA*. The resulting protein BrlA is a C_2_H_2_ zinc finger transcription factor, which plays a key role in asexual development in the pathogen *A*. *fumigatus* and the model fungus *A*. *nidulans* [35, 55]. Deletion of either *flbB* or *flbD* results in fluffy phenotypes resembling the Δ*brlA* mutant strain in *A*. *nidulans*. The FlbB impact on conidiation is similar in *A*. *fumigatus*, but the FlbD impact is less pronounced. The *flbB* deletion abolishes *flbD* expression and delays *brlA* expression [56]. In *A*. *fumigatus*, expression of the *flbD* gene requires in addition to FlbB also FlbE as further developmental regulator. Consequently, conidiation is delayed and reduced in a Δ*flbB* mutant [25, 56–58].

SomA controls *stuA* and *medA* expressions in the correlation between conidiation and adherence in *A*. *fumigatus*. StuA and MedA contribute to accurate spatial and temporal expression of *brlA* in *A*. *nidulans* [59]. Consistently, disruption of *stuA* and *medA* results in abnormal conidiophores and reduced conidiation in *A*. *fumigatus* [27–29]. The severe impairment of conidiation in the *somA* deletion mutant can be attributed to both StuA and MedA, which are also required for activating expression of genes for adherence. Uge3 is an UDP-glucose epimerase that interconverts UDP-glucose and UDP-galactose and mediates formation of galactosaminogalactan (GAG) [30]. This compound is part of the extracellular matrix and is required for biofilm formation as well as adherence and therefore plays a prominent role in pathogenesis of *A*. *fumigatus* [60]. The StuA binding sites (A/T)CGCG(T/A)N(A/C) had been defined [61] and are also present in the promoter regions, of the *uge3* (position -1651 and -1108 bp) or AFUA_3G00880 (position -3627 bp) genes for adhesion and as well in the *brlA* promoter region (position -507, -753 and -3276 bp) for asexual development (S7 Fig). This indicates that StuA has a dual role in directly activating the transcription of genes for adhesion and conidiation by binding to the corresponding promoters (Fig 9). SomA is required for the expression of *stuA* and *medA* and thereby plays an important role for conidiation as well as adhesion.

In this study, an attenuated virulence of the *Tet-somA* mutant (*Off* state) resulting in significantly reduced cellular SomA protein levels was observed in infection models with embryonated egg or with mice (Fig 8). An interesting question is which of the phenotypes which are observed in the absence of sufficient amounts of SomA protein is causing the reduction in virulence. SomA is required for the normal fungal growth rate, for asexual spore formation and for adherence. The Δ*stuA* mutant which is located downstream of the *somA* gene (Fig 9) showed abnormal impaired conidiation with a nonsignificant reduced virulence [29]. Loss of the *gpaB* gene, which is the Gα subunit of a heterotrimeric G proteins upstream of *somA* resulted in no growth retardation but attenuated virulence in a mouse model [16]. Loss of *somA* gene resulted in abolished biofilm formation and adherence to various surfaces as well as decreased *uge3* expression and GAG production (Figs 5 and 6). Gravelat *et al*. showed that adhesion is an important factor for full virulence in mice model. The deletion of *uge3* resulted in a normal growth and morphology together with an impaired adherence and reduced virulence in mice model [30]. In contrast, the Δ*ptaB* mutant showed normal virulence in mice model incorporate with reduced expression of *uge3* gene and resistance to oxidative stress (Fig 5C and S4 and S5 Figs). These data support a complex interplay of SomA with several genetic networks which ultimately has an important impact on fungal virulence in host cells (Fig 9).

The Flo8/Som1 protein family functions downstream of the cAMP/PKA signaling pathway and this pathway regulates asexual development in both *A*. *nidulans* and *A*. *fumigatus* [25]. We showed that SomA is upstream of *flbB*, *medA* and *stuA* genes (Fig 7). Overexpression of the *somA* gene in the Δ*stuA* mutant had no significant phenotype compared to the Δ*stuA* mutant. In contrast, the Δ*stuA* Δ*somA* double mutant strain resulted in a complex phenotype with more aerial hyphae and different lighter color in the back as a hint of changes in secondary metabolite production. This distinct phenotype of the Δ*stuA* Δ*somA* double deletion strain could reflect the activation of additional signal pathways. Previous studies indicated that StuA is required for conidiation and the regulation of secondary metabolite clusters [29, 35]. These studies suggested that the difference of color on the back might be due to the absence of StuA protein in the double deletion mutant. The *stuA* gene was expressed, but was reduced, in the Δ*somA* mutant (Fig 6 and S3 Fig). Macheleidt *et al*. showed that expression of *stuA* is regulated by the cAMP/PKA pathway [62]. SomA might affect the expression of the *stuA* gene but the interplay between both gene products is presumably more complex and might rather resemble a genetic network than a single signal transduction pathway (Fig 9).

There is an important interplay between conidiation and cell-cell adhesion. The formation of aerial hyphae results in vesicles, which further differentiate into spore forming cells by a polar budding process reminiscent to the yeast single cell growth mode [27, 63]. In *A*. *nidulans*, this results primarily in the formation of metulae cells, which are absent in *A*. *fumigatus* where directly the spore forming phialides are formed. Phialides are the cells, which produce the small hydrophobic non-motile conidia in a process similar to pseudohyphae formation where at the beginning the spores are attached to each other. Pseudohyphae formation in yeast requires adhesins such as Flo11, which mediate cell-cell adhesion [2]. StuA controls metulae and phialide differentiation in *A*. *nidulans* and consistently, Phd1 as homologue of StuA, governs pseudohyphal growth in *S*. *cerevisiae* [63]. Asexual spore formation also requires AbaA, which is located downstream of BrlA in the developmental cascade. Consistently, the AbaA corresponding yeast protein Tec1 is required for pseudohyphae formation. Tec1 can be replaced by *A*. *nidulans* AbaA to repair the defect of a Δ*tec1 S*. *cerevisiae* mutant strain [64].

Several proteins providing adherence in filamentous fungi have been identified. SomA affects several adhesion related genes (Fig 6), which results in the SomA mediated plastic adherence observed in this study. Hydrophobins also play a specific role in adhesion and are amphiphilic proteins involved in aerial hyphae formation and conidiation [65]. Hydrophobins as Mhp1 of the plant pathogen *Magnaporthe grisea* or Mpg1 of *M*. *oryzae* are responsible for appressorium development and subsequent entry into the plant host [66, 67]. RodA is a spore hydrophobin of *A*. *fumigatus* which prevents immune recognition and provides adherence of conidia to collagen or albumin [3]. The expression of the *rodA* gene depends on regulators as BrlA and AbaA [25] and the *rodA* expression is affected by SomA (Fig 6). Recently, galactosaminogalactan was found to be an adhesive compound produced by the *A*. *fumigatus* Uge3 epimerase, which is required for virulence [30]. We showed that SomA and PtaB regulate *uge3* expression as well as *stuA* and *medA* (Fig 6 and S4 Fig), which have been shown to control transcription of *uge3*. In addition, loss of *somA* showed reduced galactose and galactosamine production which are required for galactosaminogalactan formation (Fig 5B). Interestingly, the homologues of these genes are also present in the non-pathogenic *A*. *nidulans*. StuA, MedA and Som1 are required for normal conidiation in *A*. *nidulans* [27, 44] and StuA binding sequence is present in the promoter of the *uge3* homologue. This indicated that SomA might regulate other unknown genes required for adhesion or pathogenicity.

Most of the filamentous pathogenic fungi show host specificity and can only infect and cause disease in either plants or animals. Only a limited number of fungal pathogens such as *Aspergillus flavus* or *Fusarium oxysporum* can cause infections in both kingdoms. Plants and animals possess distinct protective systems to prevent fungal infections. This includes the plant cuticle as barrier or increased temperatures of 37°C and higher in humans [68]. Genes which are required for cuticle degradation or appressorium formation are specific for plant pathogens [69]. The set of genes from filamentous fungal pathogens, which is important for plant as well as human pathogenic fungi is limited and includes siderophores for the uptake of iron ions and adhesins as hydrophobins [70, 71]. The transcription factor Flo8/Som1 is an interesting control gene which is required for adherence, development and pathogenicity of pathogens which include filamentous fungal plant pathogens as *M*. *oryzae* as well as dimorphic and constitutively filamentous fungal human pathogens as *C*. *albicans* and *A*. *fumigatus*, respectively.

## Materials and Methods

### Strains and growth conditions

The fungal strains used in this study are listed in Table 1. The AfS35 (Δ*akuA*) was used as the background strain [72]. *A*. *fumigatus* was grown at 37°C in minimal medium (MM) as previously described [73, 74]. 1% D-glucose as carbon source and 10 mM ammonium tartrate as nitrogen source were supplemented. 2% agar and 1 mg/L pyrithiamine were used for solid medium and selection, respectively. For pyrithiamine marker recycling, 0.5% xylose was supplemented. *Escherichia coli* strain DH5α was used for construction of plasmid and was propagated in LB medium (0.5% yeast extract, 1% bacto-tryptone and 1% NaCl) at 37°C. *S*. *cerevisiae* strains BY4742, Y16870, RH2656 and RH2660 (Table 1) were used for cross-species complementation. The BY4742 (*flo8*) is derived from S288c carrying truncated *FLO8* gene [31] and RH2660 (Δ*flo8*) is derived from Σ1278b with deletion of *FLO8* gene [75, 76]. *S*. *cerevisiae* was cultivated at 30°C in either non-selective YEPD medium (1% yeast extract, 2% peptone and 2% glucose) or in SC-3 medium (0.15% yeast nitrogen base without amino acid and ammonium sulfate, 0.5% (NH_4_)_2_SO_4_, 2% glucose and 0.2% amino acid mixture lacking uracil, L-methionine and L-leucine). Appropriate amino acids were supplemented as required for adhesive assay and flocculation assay. For solid medium, 2% agar was added.

**Table 1:** Fungal strains used in this study.

Strain	Genotype	Reference
*A*. *fumigatus*		
AfS35	Δ*akuA*::*loxP*	[72]
AfGB77	Δ*akuA*::*loxP*; Δ*somA*::*six*	This study
AfGB105	Δ*akuA*::*loxP*; Δ*somA*::*somA*::*six*	This study
AfGB74	Δ*akuA*::*loxP*; *ptrA*::^*p*^ *Tet*::*somA*	This study
AfGB75	Δ*akuA*::*loxP*; *somA*::*gfp*::*ptrA*	This study
AfGB76	Δ*akuA*::*loxP*; ^*p*^ *gpdA*::*gfp*::*his2a* ^*t*^, *ptrA*	This study
AfGB106	Δ*akuA*::*loxP*; Δ*flbB*::*six*	This study
AfGB107	Δ*akuA*::*loxP*; Δ*medA*::*six*	This study
AfGB108	Δ*akuA*::*loxP*; Δ*stuA*::*six*	This study
AfGB109	Δ*akuA*::*loxP*; Δ*flbB*::*six*; ^*p*^ *gpdA*::*somA*::*his2a* ^*t*^, *ptrA*	This study
AfGB110	Δ*akuA*::*loxP*; Δ*medA*::*six*; ^*p*^ *gpdA*::*somA*::*his2a* ^*t*^, *ptrA*	This study
AfGB111	Δ*akuA*::*loxP*; Δ*stuA*::*six*; ^*p*^ *gpdA*::*somA*::*his2a* ^*t*^, *ptrA*	This study
AfGB112	Δ*akuA*::*loxP*; Δ*flbB*::*six*; Δ*somA*::*ptrA*	This study
AfGB113	Δ*akuA*::*loxP*; Δ*medA*::*six*; Δ*somA*::*ptrA*	This study
AfGB114	Δ*akuA*::*loxP*; Δ*stuA*::*six*; Δ*somA*::*ptrA*	This study
AfGB115	Δ*akuA*::*loxP*; Δ*ptaB*::*six*	This study
AfGB116	Δ*akuA*::*loxP*; Δ*ptaB*::*ptaB*::*rfp*::*six*	This study
AfGB117	Δ*akuA*::*loxP*; *somA*::*gfp*::*six*; *ptaB*::*rfp*::*ptrA*	This study
AfGB118	Δ*akuA*::loxP; *pyroA* ^*p*^ *TetOn*::*rfp*::*trpC* ^*t*^	This study
AfGB119	Δ*akuA*::*loxP*; ^*p*^ *gpdA*::*somA*::*his2a* ^*t*^, *ptrA*	This study
A1176	*pyrG1*; Δ*brlA*::*pyrG* ^+^	[55]
*S*. *cerevisiae*		
BY4742	*MATα*; *his3*Δ*1*;*leu2*Δ*0*;*lys2*Δ*0*;*ura3*Δ*0*; *flo8*	Euroscarf
Y16870	*MATα*; *his3*Δ*1*;*leu2*Δ*0*;*lys2*Δ*0*;*ura3*Δ*0*; *flo8*; Δ*flo1*::*kanMX4*	Euroscarf
RH2656	*MAT*a/ *MATα ura3-52/ura3-52*; *trp1*::*hisG/TRP1*	[75]
RH2660	RH2656; Δ*flo8*::*kanMX4/*Δ*flo8*::*kanMX4*	[75]

To determine the growth rate of the strains, 500 conidia or a portion of mycelia of the strains were inoculated in the middle of MM plates for 5 days. The colony diameters were measured every day. Doxycycline (5 mg/L) was supplemented as required.

### Nucleic acid isolation and manipulations

Recombinant DNA technologies were performed according to standard methods [77]. DNA fragments for plasmid construction, hybridization probes or sequencing were amplified by Phusion polymerase (Thermo Fisher Scientific GmbH). Primers used for plasmid construction are listed in S3 Table. Isolation of genomic DNA from *A*. *fumigatus* was performed as previously described [78]. Isolation of plasmid DNA and RNA were performed using either QIAprep Miniprep Kit (Qiagen) or RNeasy Plant Mini Kit (Qiagen) referring to user’s manual, respectively. The cDNA of *A*. *fumigatus* was generated from total RNA using the QuantiTect Reverse Transcription Kit (Qiagen) following the user’s manual.

### Deletion of *somA*

The 5’ and 3’ UTR regions were amplified with the corresponding primers HO499/500 or HO501/502. These two PCR products were fused by amplifying with the primer pair HO499/502 to yield a fragment which contains a restriction site for *Sfi*I in the middle and a restriction site for *Hin*dIII at both ends. Then it was cloned into pJET1.2 Blunt cloning vector (Fermentas GmbH). The self excising marker system, which harbors a xylose driven β-recombinase, a pyrithiamine resistance cassette and two flanking binding sites (*six*), was isolated from pSK485 [79] with *Sfi*I restriction enzyme. This recyclable marker fragment was cloned into the *Sfi*I restriction site in the previous plasmid containing fused 5’ and 3’ UTR regions to generate pME4188.

Transformations were performed as previously described to construct *A*. *fumigatus* mutants [80]. The Δ*akuA* strain (AfS35) was transformed with the deletion fragment isolated from pME4188 using *Hind*III restriction enzyme and was selected with pyrithiamine. The positive mutant (Δ*somA*::*ptrA*) was streaked out on MM plates containing 0.5% xylose and glucose to remove the pyrithiamine resistance and resulted in Δ*somA* mutant (AfGB77).

### Construction of the *somA* complemented strain

For complementation, the fragment containing 5’ UTR and *somA* gene was amplified with the primer pair HO603/601 and cloned into *Sma*I digested pUC19 (Fermentas GmbH) using the In-fusion HD Cloning Kit (Takara BioEurope/Clontech) to generate pME4189. Linear pME4189 amplified with primers HO711/611 was fused with the recyclable marker fragment and the 3’ UTR of *somA* which was amplified with the primer pair HO677/501 to yield pME4190. The complement fragment isolated from pME4190 by *Hin*dIII digestion was transformed into Δ*somA* mutant and the pyrithiamine resistance was removed to generate the complemented mutant (AfGB105).

### Construction of the *Tet-somA* strain

To overcome the defect of conidiation in the *somA* deletion mutant, a strain containing the conditional expression of *somA* gene was generated. First, the pyrithiamine resistance cassette and the Tet-On system were amplified with the primer pair HO116/675 using pCH008 [36] as template. This fragment was cloned into the linear pME4189 amplified with primers HO710/676 to yield plasmid pME4191. The Tet-On system fragment replaced 602 bp of the 5’ UTR region (position -602~-1) of *somA* gene. The fragment isolated from pME4191 using *Hin*dIII restriction enzyme was transformed into the Δ*akuA* strain to generate the *Tet-somA* mutant (AfGB74).

### Deletion of *ptaB*

The deletion of the *ptaB* gene was carried out as followed. The 5’ UTR was amplified via PCR using the primer pair PtaB-1/PtaB-2. For the 3’ UTR the primers PtaB-3/PtaB-4 were used. As selection marker the flipper cassette published by [79] was used. The cassette was received by digestion of the plasmid pSK485 with *Sfi*I. The three received fragments were integrated in the pBluescript II KS+ via an *Eco*RV restriction site. Therefore, the GeneArt Seamless Cloning and Assembly Kit (Invitrogen) was used according to user’s manual. The generated plasmid pME4361 was digested with *Pme*I and the received knock out fragment was integrated in the Δ*akuA* strain. Transformants were selected on pyrithiamine containing media and checked for correctness via Southern hybridization. To recycle the resistance cassette clones were streaked out on minimal medium containing 0.5% glucose and 0.5% xylose. The loss of the resistance cassette was checked by Southern hybridization to generate the *ptaB* knock out strain without pyrithiamine resistance (AfGB115).

### Construction of the *ptaB* complemented strain

To complement the *ptaB* deletion mutant, a fragment containing the *ptaB* gene with 5’ and 3’ UTR region was amplified with primers HO885/701 and was cloned into pUC19. The *rfp* gene and the *trpC* terminator were amplified in accordance with the primer pair HO872/873 or HO874/890 and fused by amplifying with the primer pair HO872/890 to yield a 1.5 kb fragment. This fragment was cloned into the plasmid, which contains 5’UTR-*ptaB-*3’UTR in pUC19, amplified with primers HO887/888 to yield plasmid contains *ptaB-rfp* fused gene. Afterwards, the recyclable marker was cloned into the *Sfi*I site in previous plasmid containing 5’UTR-*ptaB-rfp-trpC* ^*t*^-3’UTR to generate pME4362. For complementation, the Δ*ptaB* mutant was transformed with the fragment isolated from pME4362 with *Hin*dIII restriction enzyme to generate the complemented strain (AfGB116).

### Construction of Δ*medA*, Δ*stuA* and Δ*flbB* mutants and derivatives

To construct plasmid pME4359, the 5’ and 3’ UTR regions of the *medA* gene were amplified with the corresponding primers HO852/881 or HO854/855 and fused by HO852/855. The fused fragment was further cloned into pUC19. The recyclable marker was cloned into the plasmid, which contains used 5’ and 3’ UTR of *medA*, amplified with primers HO854/881 to generate pME4359. The construction of plasmids pME4360 and pME4358 was similar to pME4359. The 5’ and 3’ UTR regions of *stuA* were amplified with primer pairs HO848/880 and HO850/851. These two PCR products were fused with primer HO848/851, cloned into pUC19 and linked with recyclable marker to yield pME4360. For pME4358, two UTR regions of *flbB* were amplified with primers HO844/879 and HO846/847 and fused by HO 844/847. The fused PCR was cloned into pUC19. The recyclable marker was cloned into the plasmid, which contains fused UTRs of *flbB*, amplified with primers HO882/846 to generate pME4358. The Δ*akuA* strain was transformed with the deletion fragment isolated from pME4359, pME4360 or pME4358 using *Hin*dIII and *Apa*LI restriction enzymes to obtain Δ*medA;* Δ*akuA* (AfGB107), Δ*stuA;* Δ*akuA* (AfGB108) or Δ*flbB;* Δ*akuA* (AfGB106) mutants.

For construction of the *somA* overexpression plasmid, the *somA* gene was amplified with primer pair HO531/532 and cloned into *Pme*I site in pSK379 to yield pME4363. Plasmid pME4363 was transformed into the Δ*akuA* background, Δ*medA*, Δ*stuA* or Δ*flbB* mutants to generate the OE*somA* (AfGB119), Δ*medA;*OE*somA* (AfGB110), Δ*stuA;*OE*somA* (AfGB111) or Δ*flbB;*OE*somA* (AfGB109) strains, respectively. The *somA* gene is ectopical overexpression. Double deletion strains Δ*medA;* Δ*somA* (AfGB113), Δ*stuA;* Δ*somA* (AfGB114) or Δ*flbB;* Δ*somA* (AfGB112) were constructed by transformation of the deletion fragment isolated from pME4188 using *Hin*dIII restriction enzyme into the corresponding strain Δ*medA*, Δ*stuA* or Δ*flbB* mutant.

### Southern hybridization

All mutants were confirmed by Southern hybridization which was performed as described previously [81]. Preparation of probes was carried out using AlkPhos Direct Labelling Reagents Kit (GE Healthcare) according to user’s manual. Detection of probes was performed with the CDP-Star Detection reagent (GE Healthcare) following user’s manual.

### Computational analysis

Blast searches and protein conserved domain identification were conducted at the National Center for Biotechnology Information (http://www.ncbi.nlm.nih.gov). Protein alignments were made by Clustal Omega at European Molecular Biology Laboratory–European Bioinformatics Institute (http://www.ebi.ac.uk). Nuclear localization signal (NLS) was predicted at cNLS mapper (http://nls-mapper.iab.keio.ac.jp/). The protein name and gene number of *A*. *fumigatus* are according to the AspGD (http://www.aspergillusgenomes.org) [82]. Protein and DNA sequence analysis was performed using Lasergene software (Dna Star Inc., Madison, WI, USA).

### Yeast complementation

To complement *flo8-*deficient yeasts, two SomA cDNA variants were amplified with primers HO441/HO442, digested with *Spe*I/*Hin*dIII and cloned into pME2786 and pME2787 to generate plasmids pME4192, pME4193, pME4194 and pME4195, respectively (Table 2). For positive control, *S*. *cerevisiae* Flo8 (*FLO8*) was amplified with primers HO446/447 and cloned into *Sma*I digested pME2786 and pME2787 to generate plasmids pME4196 and pME4197, respectively. Empty plasmids pME2786 and pME2787 were used as negative control. Transformation of *S*. *cerevisiae* was performed as described previously [83]. The pME2786 based plasmids were transformed into haploid strain BY4742 and selected on SC-Leu medium; meanwhile, the pME2787 based plasmids were transformed into haploid strain BY4742 and diploid strain RH2656 and RH2660 using selection medium SC-Ura.

**Table 2:** Plasmids used in this study.

Plasmid	Description	Reference
*A*. *fumigatus*		
pJET 1.2/blunt	Cloning vector	(Fermentas)
pUC19	Cloning vector	(Fermentas)
pBluescript II KS+	Cloning vector	(Stratagene)
pCH008	Plasmid contains Tet-On system and ptrA marker	[36]
pME4435	^*p*^ *gpdA*::*gfp*::*his2a* ^*t*^::*ptrA* in pSK379	This study
pME4188	Deletion cassette *somA* in pJET1.2	This study
pME4189	*5’ UTR*::*somA* in pUC19	This study
pME4190	Complement cassette *somA* in pUC19	This study
pME4191	*5’ UTR*::*ptrA*::^*p*^ *Tet*::*somA* in pUC19	This study
pME4198	*5’ UTR*::*somA*::*gfp*::*ptrA*::*3’ UTR* in pUC19	This study
pME4358	Deletion cassette *flbB* in pUC19	This study
pME4359	Deletion cassette *medA* in pUC19	This study
pME4360	Deletion cassette *stuA* in pUC19	This study
pME4361	Deletion cassette *ptaB* in pBluescript KSII+	This study
pME4362	*5’ UTR*::*ptaB*::*rfp*::*trpC* ^*t*^::*ptrA*::*3’ UTR* in pUC19	This study
pME4363	Overexpression of *somA* in pSK379	This study
pSK379	Plasmid contains ^*p*^ *gpdA*::*his2a* ^*t*^::*ptrA*	[79]
pSK485	Plasmid contains recyclable marker driven by xylose promoter	[79]
*S*. *cerevisiae*		
pME2786	pRS425 containing *MET25* promoter *CYC1* terminator, *LEU2*, *20μm*, *Amp*, *ori*	[93]
pME2787	pRS426 containing *MET25* promoter *CYC1* terminator, *URA3*, *2μm*, *Amp*, *ori*	[93]
pME2167	3 kb *FLO11* promoter in YEp355	[34]
p*FLO11-2/1* to p*FLO11-15/14*	400 bp *FLO11* promoter sequence fragments cloned into pLG669Z	[34]
pME4192	*somA* cDNA in pME2786	This study
pME4193	*somA* cDNA variant in pME2786	This study
pME4194	*somA* cDNA in pME2787	This study
pME4195	*somA* cDNA variant in pME2787	This study
pME4196	*FLO8* in pME2786	This study
pME4197	*FLO8* in pME2787	This study

For haploid adhesion assays, the transformed strains were grown on corresponding selective plates for 3 days at 30°C, and then those plates were washed with water until the negative control was washed away. The plates were photographed before and after washing. The flocculation assay in haploid yeasts was performed as previously described [33]. Briefly, the transformed BY4742 strains were inoculated in either SC-Leu or SC-Ura liquid medium and incubated for one day at 30°C. Following, 1 mL of EDTA (0.5 M, pH8) was added and the samples were vortexed until flocculation were disrupted. The value of flocculation was determined by F = 1-B/A, where A is OD_600_ in solution without 0.1% CaCl_2_ and B is OD_600_ in the presence of 0.1% CaCl_2_. For pseudohyphal growth, diploid transformants were grown on SLAD (synthetic low ammonium dextrose medium) for 6 days at 30°C, and then the plates were photographed.

### Analysis of *FLO11* promoter elements and promoter binding

To verify that SomA could act on the *FLO11* promoter, both pME4192 and pME4193 (Table 2) were co-transformed with plasmid pME2167 which contains 3 kb of the *FLO11* promoter fused with *LacZ* reporter gene, in Y16870 strain (*flo8/*Δ*flo1*) and transformants were selected on SC-Ura-Leu medium. Transformations of plasmid pME2167 with either *FLO8* (pME4196) or empty vector (pME2786) in Y16870 strain were used as positive or negative control, respectively. To identify the specific DNA motif that was regulated by SomA and Flo8, 14 reporter constructs containing 400 bp *FLO11* promoter fragments which have 200 bp overlaps and were cloned in front of *CYC1*::*LacZ* fused gene [34]. These 14 reporter plasmids (p*FLO11-2/1* to p*FLO11-15/14*) were co-transformed with plasmids pME4192, pME4193 or pME4196 in Y16870 and selected with SC-Ura-Leu medium. The regulation of SomA and Flo8 on the *FLO11* promoter was determined by β-galactosidase assays.

### β-galactosidase activity assays

The assays were performed as previously described [75]. Yeast strains were grown in SC-Ura-Leu liquid medium overnight as pre-culture, 1 mL of the pre-culture was added to 10 ml of SC-Ura-Leu-Met liquid medium as main-culture for 6 h. β-galactosidase activities were calculated by following formula (OD_420_ × 0.35) / (0.0045 × protein concentration × extract volume × time) [84]. Protein concentrations were determined by OD_595_ with Bradford assay [85].

### Microscopy analysis

To analyze mycelial morphology, strains were grown on agar-coated slides with thin layer of MM agar for 28 h at 37°C and then were observed under the Olympus Axiolab microscope with 400-fold magnification. For aerial hyphae visualization, strains were grown on agar slides with either MM agar or MM agar containing 5 mg/L doxycycline for 28 h at 37°C, and were observed under the microscope as well.

### Construction of *somA-gfp* and *somA-gfp/ptaB-rfp* strains

To construct SomA-GFP tagged protein, *gfp* gene was amplified with primers HO210/713 from pME4435. The 3’ UTR region of *somA* gene was amplified with primer set HO648/677. This fragment and *gfp* fragment were cloned into linear pME4189 containing 5’UTR-*somA* gene amplified with primers HO611/712 using In-fusion Kit resulting plasmid carrying *somA*-*gfp* fused gene together with 5’ and 3’ UTR regions. Then, the recyclable marker fragment was cloned into previous plasmid amplified with HO697/501 to yield pME4198. The Δ*akuA* background strain was transformed with *somA-gfp* fused fragment which was isolated from pME4198 using *Hin*dIII restriction enzyme and selected by pyrithiamine to yield *somA-gfp* strain (AfGB65). The Δ*akuA* strain was transformed with pME4435 containing overexression *gfp* for GFP-Trap control. To perform the co-immunoprecipitation experiments, the fragment isolated from pME4362 with *Hin*dIII restrict enzyme was transformed into the *somA-gfp* mutant and resulted in *ptaB-rfp/somA-gfp* strain (AfGB117).

### Immunoprecipitation and Trypsin digestion

A GFP overexpression strain and two independent SomA-GFP strains were grown in 200 mL of MM for 24 h at 37°C. Total proteins were extracted by mixing grinded mycelia with B* buffer (300 mM NaCl, 100mM Tris pH 7.0, 10% glycerol, 2 mM EDTA, 0.02% NP40, 2 mM DTT, 1 mM PMSF, 2 protease inhibitor pills/1 mL (Complete, EDTA-free, Roche)). The GFP trapping was performed by following steps: The crude protein extracts were mixed with 15 μL GFP-Trap beads (ChromoTek) which has been washed with B* buffer and incubated for 2 h on a rotating machine at 4°C. After the incubation, the beads were washed twice with 1.5 mL and 1 mL of B* buffer. After the centrifugation for 1 min at 4500 rpm at 4°C, the supernatant was removed. The beads were resuspended with 40 μL 6 X loading dye (250 mM Tris pH 6.8, 15% β-mercaptoethanol, 30% glycerol, 7% SDS, 0.3% bromophenol blue) and were boiled for 6–8 min at 95°C to separate the proteins from the beads. The eluted protein were applied to 12% SDS-PAGE. Gel pieces were isolated and performed trypsin digestion. Trypsin digestion was performed as previously described [86, 87].

The *somA-gfp/ptaB-rfp* strain (AfGB117), GFP (AfGB76) or RFP overexpression (AfGB118) strains were grown in 1 L of MM for 24 h at 37°C for co-immunoprecipitation experiment. Protein extraction and immunoprecipitation were performed as previous described. RFP-Trap beads (ChromoTek) was used for the RFP trapping.

### Western experiments

20 μg of eluted protein from co-immunoprecipitations were subjected to 12% SDS-PAGE after heating in SDS loading dye at 95°C for 10 min and were transferred to a nitrocellulose membrane (Whatman). The enhanced chemiluminescence method was used for detection as previous described [88]. Signals were recorded with a Fusion-SL7 detection system (Peqlab) and the Bio 1D software (Peqlab). Detection of GFP or RFP fused proteins was carried out using α-GFP (Santa Cruz) or α-RFP antibody (ChromoTek), respectively. UbiA antibody (Genescript) was used for ubiquitin detection. The horseradish peroxidase-coupled rabbit (Invitrogen) or mouse antibody (Jackson ImmunoResearch) were used as secondary antibodies.

### Mass spectrometry analysis and database searching

Mass spectrometry analysis was performed as previously described [87, 89]. Orbitrap raw files were analyzed with the Proteome Discoverer 1.4 software (Thermo Scientific, San Jose, Ca, USA) using the Mascot and SequestHT search engines with an *A*. *fumigatus* protein database with the following criteria: Peptide mass tolerance 10 ppm; MS/MS ion mass tolerance 0.8 Da, and up to two missed cleavages allowed. The variable modification considered was methionine oxidation, and carbamidomethylation was considered as fixed modification. High peptide confidence and a minimum of two peptides per protein were used as result filters. Heatmaps of MaxQuant results (version 1.4.1.2 v) were made with Perseus (version 1.4.1.3 v) software [90].

### Adherence assays

Two adherence assays were performed as previous described [25]. For biofilm formation 1 mL of Sabouraud broth containing 10^5^ conidia of strains were inoculated into 6-well Nunclon Δ surface culture plates (Nunc) at 37°C for 24 h and 5 mg/L doxycycline was supplemented in the medium of indicated strains. Afterwards, the culture medium was removed and the wells were washed three times with PBS. Biofilm was visualized by applying 2 mL of 0.1% crystal violet solution for 5 min. Excess stain was removed and the plates were washed three time with 3 mL of sterile water. The remained stain in biofilm was extracted by adding 1 mL of ethanol. The biofilm density was determined by measuring the absorbance of the destained solution at 570 nm. The biofilm formation assays were performed in triplicate and experiments were repeated 3 times.

To test the adherence of mutants to plastic and fibronectin, 10^4^ conidia in 1 mL of Sabouraud broth were incubated at 37°C for 8 h for germination and 5 mg/L doxycycline were supplemented in the medium of the indicated strains. The 6-well culture plates were untreated or coated with 0.01 mg/mL of fibronectin overnight and were inoculated with 150 germlings of the mutants and incubated at 37°C for 30 min. Afterwards, wells were washed 3 times with 3 mL of PBS and covered with YEPD agar. Fungal colony was quantified after incubation at 37°C. The adherence assay was performed in triplicates and experiments were repeated 3 times.

### Quantitative real-time PCR analysis

Quantitative real-time PCR (qRT-PCR) was performed with MESA GREEN qPCR MasterMix Plus for SYBR (Eurogentec) using CFX Connect Real-Time PCR system (Bio-Rad). The Δ*akuA* strain and mutants were grown in liquid MM medium for 20 h at 37°C and RNA was extracted using RNeasy Plant Mini Kit (Qiagen). DNase digestion and subsequent cDNA synthesis was carried out using 0.8 μg of RNA with the QuantiTect Reverse Transcription Kit (Qiagen). To perform the shift experiments, the indicated strains grown on liquied MM medium for 18 h at 37°C. Afterwards, the mycelium were shifted to liquid MM medium for 8 h or solid MM plate for 24 h. Addition of Doxycycline (5 mg/L) was noted. Each sample was performed in duplicates and the experiment was repeated two times. The histone H2A (3G05360) was used as reference gene for normalization. The relative expression of the gene of interest was calculated using the ΔΔCT method as previously described [91]. All the primers used for quantitative real-time PCR were listed in S3 Table.

### Galactosaminogalactan (GAG) assay

The assay was performed as previous described with modification [30, 92]. 5*10^7^ spores of the strain *Tet-somA* were inoculated in 100 ml modified Brian medium with 50 μg/ml doxycycline and grown for 20 hours at 37°C. The mycelium was shifted to 100 ml modified Brian medium with and without doxycycline. After 24 hours of growth at 37°C, the culture supernatants were precipitated with 2.5 volumes of ethanol. The pellets were washed with 150 mM NaCl and extracted with 8 M urea. The supernatants were dialyzed against water and dried. Polysaccharides were hydrolyzed (4 M TFA, 100°C, 4 h) and the obtained monosaccharides were derivatized with MSTFA and measured in Gas chromatography-mass spectrometry (GC-MS) (Varian CP-Sil8 CB for amines (30 m, 0.25 mm, 0.25 μm)).

### Stress test

Fresh harvest spores were counted and adjusted to 10^6^ spores/ml. Approximately 2000 spores were spotted on minimal medium plates and incubated for three days at 37°C. For inducing cell wall stress conditions SDS was used at a final concentration of 0.01%. For oxidative stress H_2_O_2_ was used at a final concentration of 2 mM and 3 mM, respectively

### Ethics statement

Mice were cared for in accordance with the principles outlined by the European Convention for the Protection of Vertebrate Animals Used for Experimental and Other Scientific Purposes. According to that, chicken embryo is not animal (http://conventions.coe.int/Treaty/en/Treaties/Html/123.htm). All animal experiments were in compliance with the German animal protection law and were approved by the responsible Federal State authority “Thüringer Landesamt für Lebensmittelsicherheit und Verbraucherschutz” and ethics committee “Beratende Komission nach § 15 Abs. 1 Tierschutzgesetz” with the permit Reg.-Nr. 03-001/12. Fertilized chicken eggs were obtained from a local producer and stored at 4°C for maximum of 4 days before incubation. Embryonation was performed by incubation of the fertilized eggs in an incubator with 60% humidity at 37°C in the lab. The embryonated eggs were infected with spores on day 10 of embryonation and the infection experiments was terminated on day 17 of embryonation.

### Egg infection model

Egg infection model was performed as described previously [40]. Eggs were incubated in an incubator with 60% humidity for 10 days at 37°C. Each *A*. *fumigatus* strain was grown on malt extract agar (Oxoid) with 5 mg/L doxycycline for 7 days and the conidia were harvested freshly on the day for infection. Each egg was inoculated with 1000 conidia in 100 μL PBS with 5 mg/L doxycycline (corresponding to the volume of the egg) and 20 eggs were inoculated for one strain. Doxycycline was not added to the PBS solution in the *Tet-somA* (*Off* state) and the complemented strain. The embryonic death was determined by the loss of movement. Survival data were plotted by Kaplan–Meier curves and statistically analyzed by log rank test using Graph Pad Prism 5.00 (GraphPad Software).

### Mouse infection model

Virulence of *A*. *fumigatus* mutant strains was tested in an established murine model for invasive pulmonary aspergillosis [41]. To induce neutropenia in female CD-1 mice (Charles River), cyclophosphamide (140 mg/kg; Sigma-Aldrich) was injected intraperitoneally on days -4, -1, 2, 5, 8 and 11, with an additional subcutaneous dose of cortisone acetate (200 mg/kg) on day -1. Mice were anaesthetized and intranasally infected with 20 μL of a fresh suspension containing 2 x 10^5^ conidia. A control group was mock-infected with PBS.

The health status was monitored twice a day for 14 days and moribund animals, defined by severe dyspnoea, severe lethargy, or weight loss > 20%, were sacrificed. Infections were performed with a group of 10 mice for each tested strain. A control group of 5 mice was not infected (inhaled PBS). Survival data were plotted by Kaplan–Meier curves and statistically analyzed by log rank test and Gehan-Breslow-Wilcoxon test using Graph Pad Prism 5.00 (GraphPad Software).

Lungs from sacrificed animals were fixed in formalin and paraffin-embedded for histopathological analyses according to standard protocols. 4-μm sections were stained using Periodic acid-Schiff (PAS, hyphae stained pink). Sections were analyzed with the Zeiss Axio Imager.M2 microscope and images were taken with a Zeiss Axiocam 105 color camera and analyzed by Zen 2012 software (Zeiss).

### Accession number

*S*. *cerevisiae*: Flo8, P40068; Mfg1, Q07684; Mss11, Q03825; Flo11, P08640.

*A*. *fumigatus*: SomA, Q4WAR8; PtaB, Q4X0N1; BrlA, Q4WRE4; RodA, P41746; FlbB, Q4X053; FlbC, Q4X0E3; FlbD, Q4WK99; StuA, Q4X228; MedA, Q4X0J5; Uge3, Q4WX18; *AfuA_3g13110*, Q4WYI0; *AfuA_3g00880*, Q4WFV6; VelC, Q4WPM3.

## Supporting Information

S1 Fig Loss of *ptaB* resulted in slow growth and delay conidiation. Colony morphology and growth rate of the indicated strains. All strains were grown on MM plate for 5 days at 37°C. Values in the graph are indicated as means ± standard error.
(TIF)

S2 Fig Addition of doxycycline has no effect on the gene expression in the Δ*akuA* strain. Relative expression of genes encoding proteins that regulate conidiation and biofilm formation in the Δ*akuA* strain. The strain was cultivated in liquid MM medium for 18 h at 37°C and shifted to solid MM plate for 24 h at 37°C. Addition of 5 mg/L doxycycline is indicated as (+). Levels for the Δ*akuA* strain without the drug were set to 1 Graph indicates mean ± standard errors from two independent experiments.
(TIF)

S3 Fig The Δ*somA* mutant showed reduced expression of development and adherence associated genes. Relative expression of genes encoding proteins that regulate conidiation and adhesion in the indicated strains. The strains was cultivated in liquid MM medium for 20 h at 37°C. Graph indicates mean ± standard errors from two independent experiments.
(TIF)

S4 Fig PtaB controls expression of development and biofilm formation related genes. Relative expression of genes encoding proteins that regulate conidiation and biofilm formation in the indicated strains. All strains was cultivated in (A) liquid MM medium for 20 h at 37°C and shifted to (B) solid MM plate for 24 h at 37°C. Graph indicates mean ± standard errors from two independent experiments.
(TIF)

S5 Fig The *ptaB* deletion strain shows increased resistance to oxidative stress. Growth test of the indicated strains. 2000 spores of each strain were spotted on minimal plates (MM) with and without H_2_O_2_ and SDS respectively. Plates were incubated for three days at 37°C.
(TIF)

S6 Fig Western hybridization of SomA-GFP and PtaB-RFP ubiquitination. Western hybridization of GFP-Trap and RFP-Trap enrichments with α-UbiA antibody. The strains were grown in MM medium for 24 h at 37°C. Protein extracts were performed with either GFP-Trap or RFP-Trap beads and the eluted proteins were separated by 12% SDS-PAGE.
(TIF)

S7 Fig Putative StuA DNA binding sites in conidia control and adhesion-associated genes of *A*. *fumigatus*. Positions of StuA binding sites (A/T)CGCG(T/A)N(A/C) [61] in 3.5 kb promoter of *brlA*, *uge3* and *3g00880* genes.
(TIF)

S1 Table LC/MS raw data (XLSX)

S2 Table Analyzed LC/MS data (XLSX)

S3 Table Primers used in this study (DOCX)
