# Supplementary figures and images for: Transcription Factor SomA Is Required for Adhesion, Development and Virulence of the Human Pathogen Aspergillus fumigatus
Source: PLoS Pathog. 2015 Nov 3;11(11):e1005205. doi: 10.1371/journal.ppat.1005205 (PMC4631450; doi:10.1371/journal.ppat.1005205)

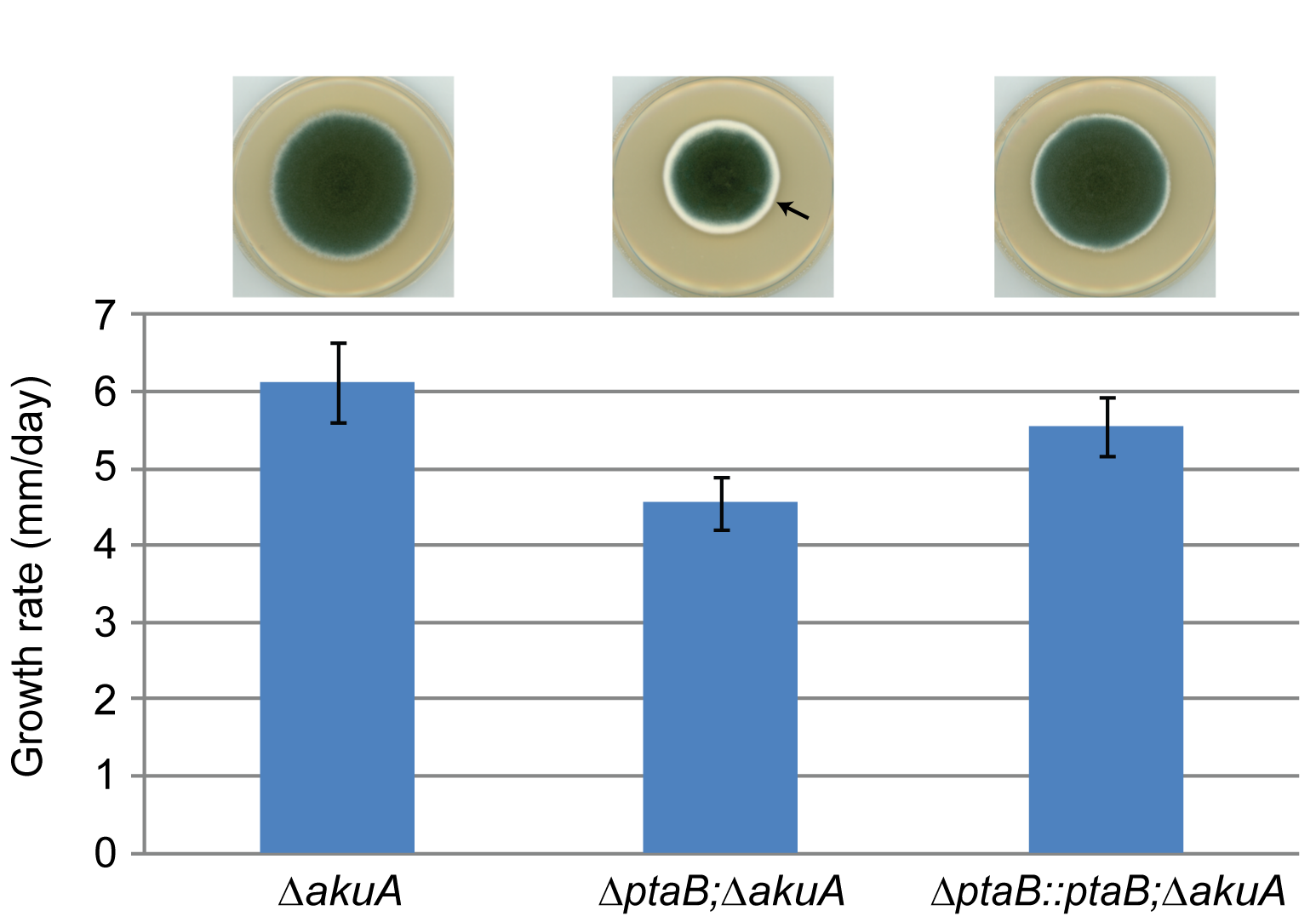

Supplement: S1 Fig — Colony morphology and growth rate of the indicated strains. All strains were grown on MM plate for 5 days at 37°C. Values in the graph are indicated as means ± standard error. (TIF) [file ppat.1005205.s001.tif]

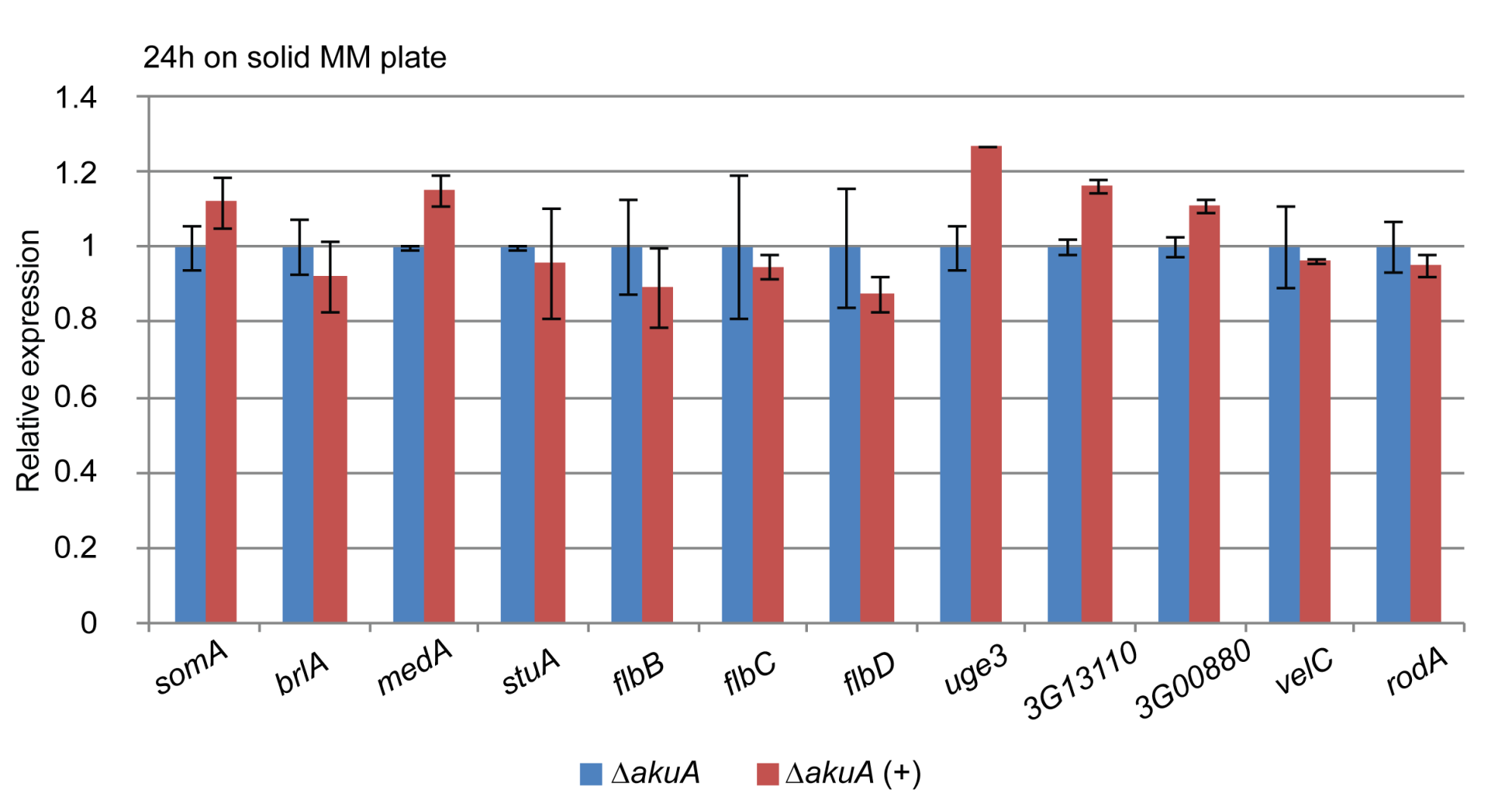

Supplement: S2 Fig — Relative expression of genes encoding proteins that regulate conidiation and biofilm formation in the ΔakuA strain. The strain was cultivated in liquid MM medium for 18 h at 37°C and shifted to solid MM plate for 24 h at 37°C. Addition of 5 mg/L doxycycline is indicated as (+). Levels for the ΔakuA strain without the drug were set to 1 Graph indicates mean ± standard errors from two independent experiments. (TIF) [file ppat.1005205.s002.tif]

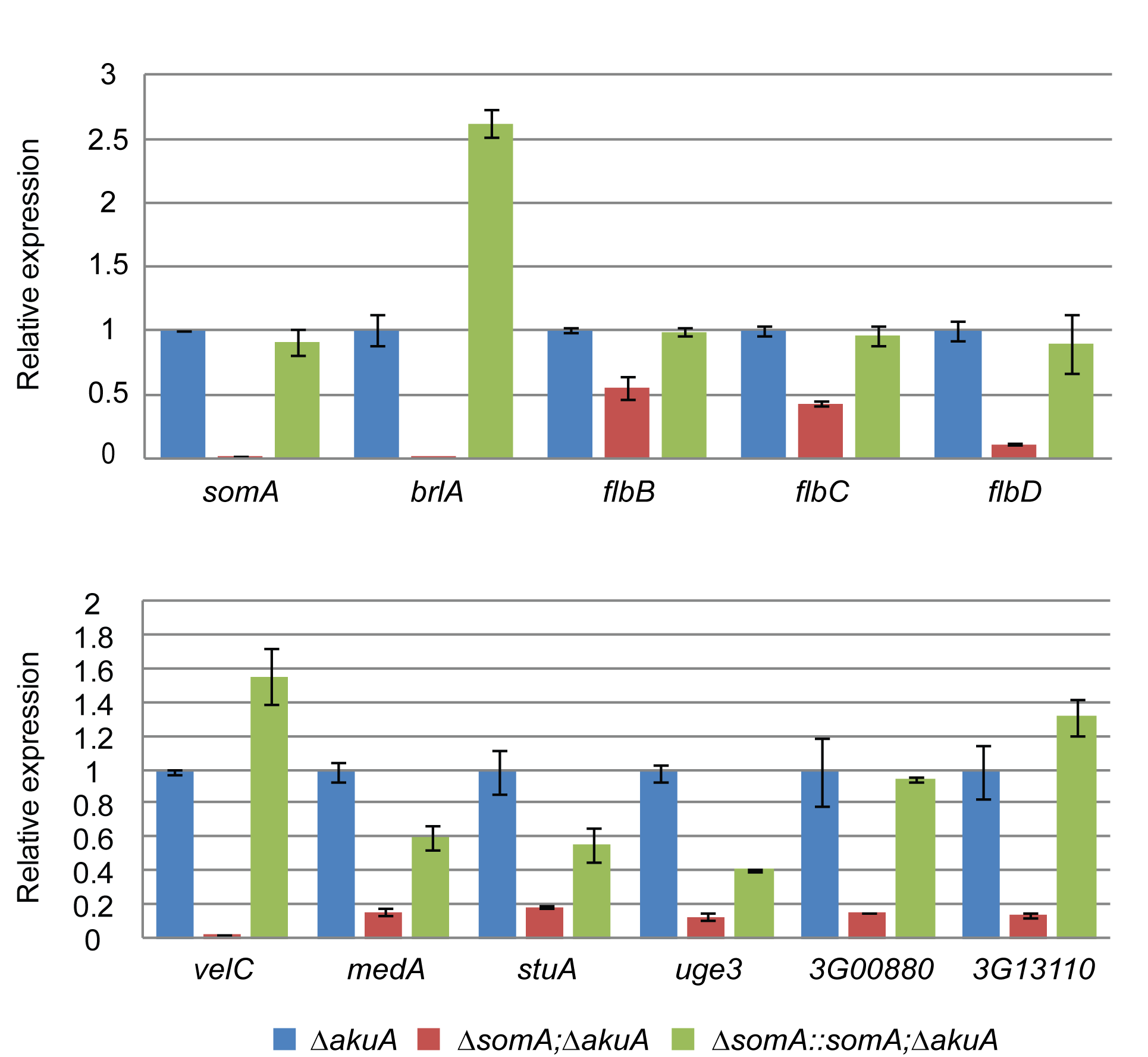

Supplement: S3 Fig — Relative expression of genes encoding proteins that regulate conidiation and adhesion in the indicated strains. The strains was cultivated in liquid MM medium for 20 h at 37°C. Graph indicates mean ± standard errors from two independent experiments. (TIF) [file ppat.1005205.s003.tif]

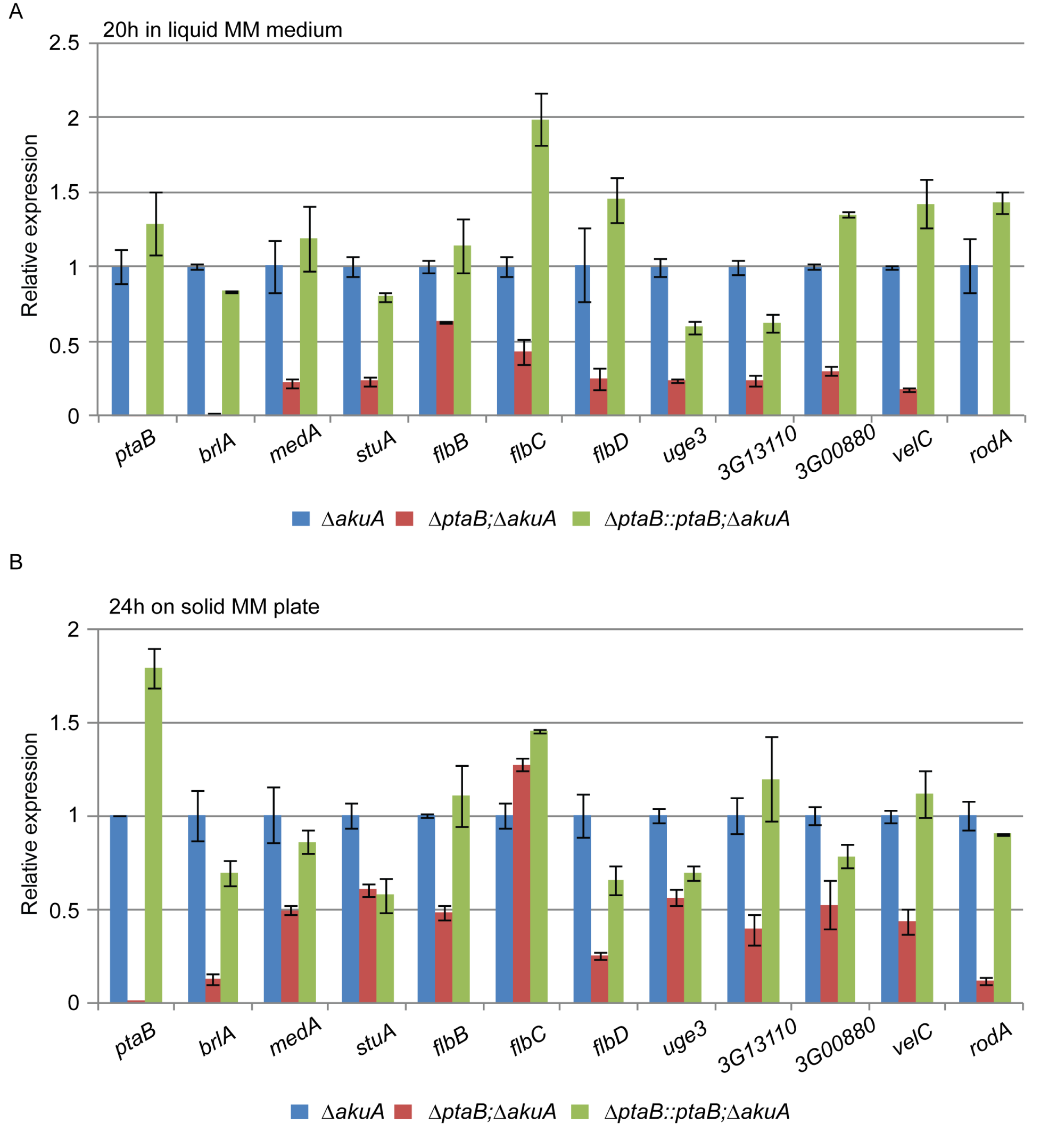

Supplement: S4 Fig — Relative expression of genes encoding proteins that regulate conidiation and biofilm formation in the indicated strains. All strains was cultivated in (A) liquid MM medium for 20 h at 37°C and shifted to (B) solid MM plate for 24 h at 37°C. Graph indicates mean ± standard errors from two independent experiments. (TIF) [file ppat.1005205.s004.tif]

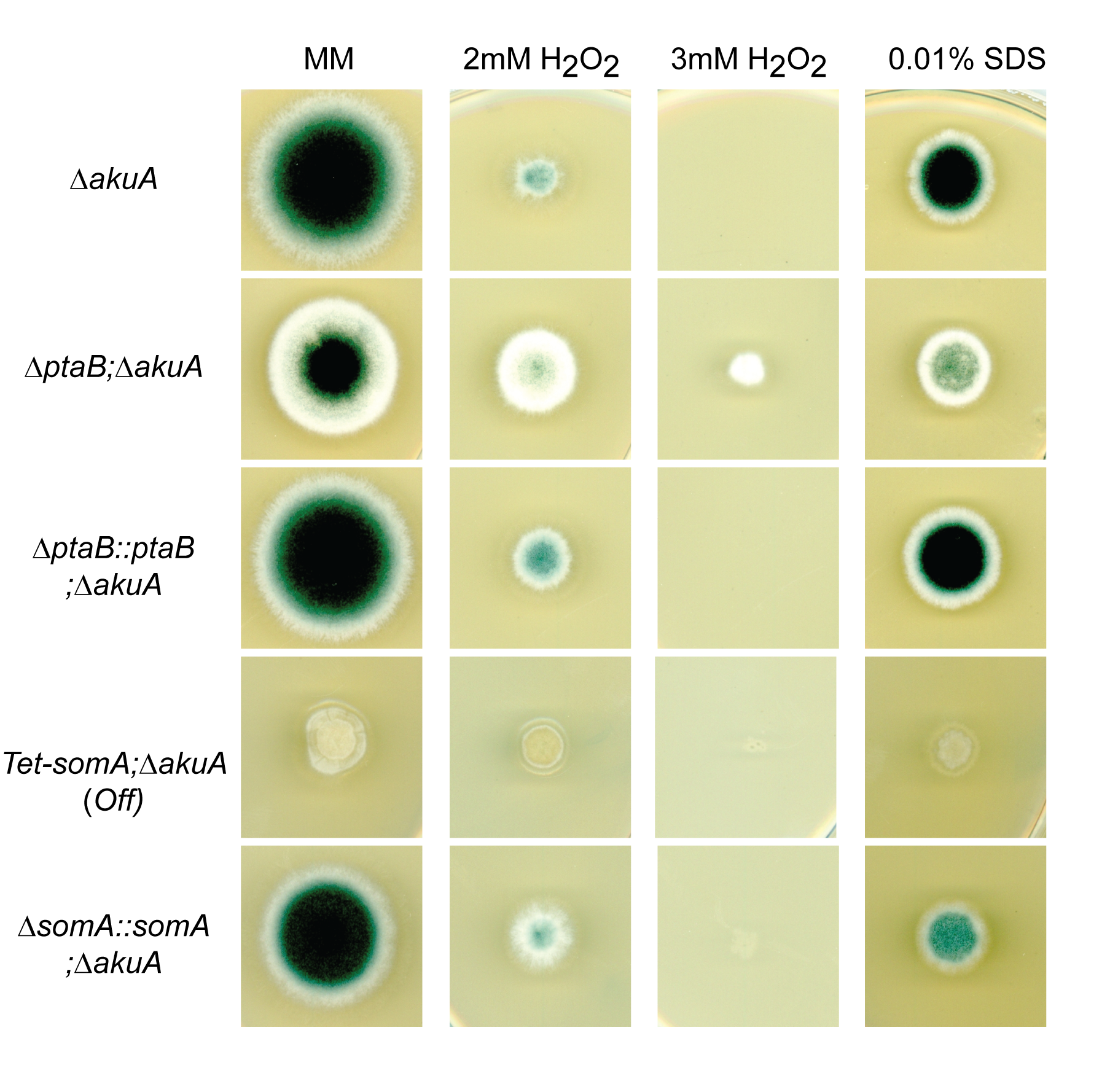

Supplement: S5 Fig — Growth test of the indicated strains. 2000 spores of each strain were spotted on minimal plates (MM) with and without H2O2 and SDS respectively. Plates were incubated for three days at 37°C. (TIF) [file ppat.1005205.s005.tif]

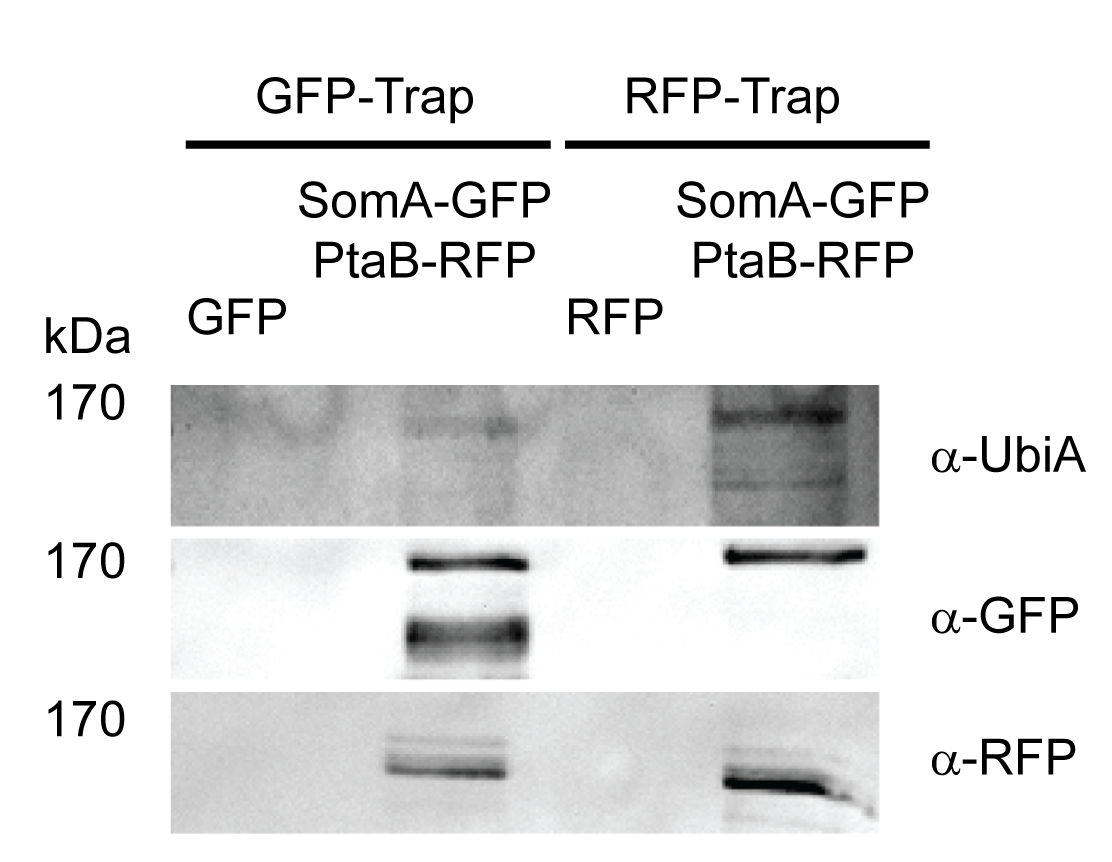

Supplement: S6 Fig — Western hybridization of GFP-Trap and RFP-Trap enrichments with α-UbiA antibody. The strains were grown in MM medium for 24 h at 37°C. Protein extracts were performed with either GFP-Trap or RFP-Trap beads and the eluted proteins were separated by 12% SDS-PAGE. (TIF) [file ppat.1005205.s006.tif]

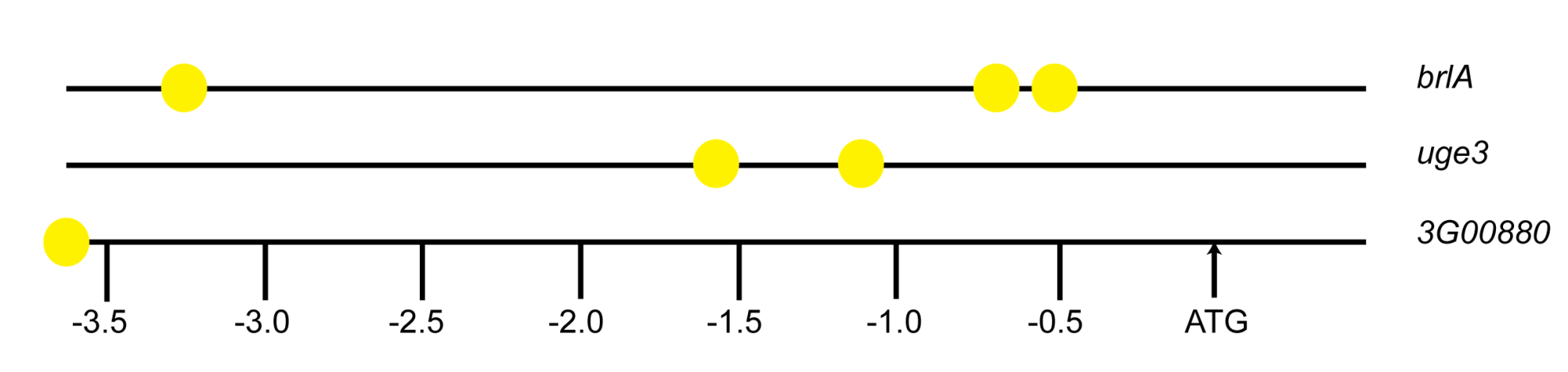

Supplement: S7 Fig — Positions of StuA binding sites (A/T)CGCG(T/A)N(A/C) [61] in 3.5 kb promoter of brlA, uge3 and 3g00880 genes. (TIF) [file ppat.1005205.s007.tif]
