# Supplementary material for: Transcription Factor SomA Is Required for Adhesion, Development and Virulence of the Human Pathogen Aspergillus fumigatus
Source: PLoS Pathog. 2015 Nov 3;11(11):e1005205. doi: 10.1371/journal.ppat.1005205 (PMC4631450; doi:10.1371/journal.ppat.1005205)
Supplement: S3 Table — (DOCX) [file ppat.1005205.s010.docx]

**S3 Table. Primers used in this study.**

| Primer | Sequence (5’→3’) | Description |
| --- | --- | --- |
| HO441 | ACTAGTATGAATCAGATGAATGTGACGGGG | *somA* with *Spe*I site |
| HO442 | AAGCTTCTGAAGAACCGACGGACTCATTTA | *somA* with *Hin*dIII site |
| HO446 | GGATCCATGAGTTATAAAGTGAATAGTTCGTATCC | *FLO8* |
| HO447 | CTCGAGGACTTCAGCCTTCCCAATTAATAAA | *FLO8* |
| HO499 | AAGCTTCCGAACAAGCGATTTACGCC | *somA* 5' UTR with *Hin*dIII site |
| HO500 | GACCTATAGGCCTGAGGGTGGCACTGCGAGGAGTTT | *somA* 5' UTR with *Sfi*I site |
| HO501 | CATAATATGGCCATCTAATGAGTCCGTCGGTTCTTCAGTT | *somA* 3' UTR with *Sfi*I site |
| HO502 | AAGCTTAAGATAGGCTGTCAGGATTGTACGG | *somA* 3' UTR with *Hin*dIII site |
| HO601 | CTCTAGAGGATCCCCTGAAGAACCGACGGACTCATTTA | *somA* with pUC19 overhang |
| HO603 | TCGAGCTCGGTACCCAAGCTTCCTTCAAAGATAACCCCTA | *somA* 5' UTR with pUC19 overhang |
| HO611 | GGGGATCCTCTAGAGTTAACCTGCAG | pUC19 |
| HO711 | TTGACCTATAGGCCTTTATAAGCCATCTCCGGCGC | *somA* with *Sfi*I site |
| HO677 | CTCTAGAGGATCCCCAAGATAGGCTGTCAGGATTGTACGG | *somA* 3' UTR with pUC19 overhang |
| HO116 | TCTTGCATCTTTGTTTGTATTATACTGTC | *ptrA* |
| HO675 | GGTGATGTCTGCTCAAGCGG | Tet-On |
| HO676 | GCTTGAGCAGACATCACCATGAATCAGATGAATGTGACGGG | *somA* with Tet-On overhang |
| HO710 | AACAAAGATGCAAGATTCGATGGGCGACACGAA | *somA* 5' UTR with *ptrA* overhang |
| HO210 | CTACTTGTACAGTTCGTCCAT | *gfp* |
| HO713 | GGAGGATCAGGAGGAATGGTGAGCAAGGGCGAGGAGCTG | *gfp* with linker |
| HO648 | GAACTGTACAAGTAGAATGAGTCCGTCGGTTCTTCAGTT | *somA* 3' UTR with *gfp* overhang |
| HO697 | TTGACCTATAGGCCTCTACTTGTACAGTTCGTCCAT | *gfp* with *Sfi*I site |
| HO712 | TCCTCCTGATCCTCCTAAGCCATCTCCGGCGCC | *somA* with linker |
| HO885 | TCGAGCTCGGTACCCAAGCTTCAGAGGTGACCTGATCTGTTAGCA | *ptaB* 5’ UTR with pUC19 overhang |
| HO701 | CTCTAGAGGATCCCCTGTAGTGAGGTGGGATGGCGTT | *ptaB* 3’ UTR with pUC19 overhang |

**Table S2. Continued.**

| Primer | Sequence (5’→3’) | Description |
| --- | --- | --- |
| HO872 | GGTGGTAGCGGTGGTATGGTCAGCAAGGGCGAAGAG | *rfp* |
| HO873 | TCACTTGTACAGCTCGTCCATGCC | *rfp* |
| HO874 | GAGCTGTACAAGTGAGGATCCCCGACGCCGAC | *trpC* terminator |
| HO890 | GGCCTAGATGGCCAAGAAGGATTACCTCTAAACAAGTGTACCTGTG | *trpC* terminator |
| HO887 | gaccataccaccgctaccaccTGCAGTACCCTTCTGTCTTTTCCCAC | *ptaB* with *rfp* overhang |
| HO888 | ttggccatctaggccGAGATGGTTTCCGTTTTCGCTTGG | *ptaB* 3’ UTR with *Sfi*I overhang |
| HO531 | GTTTAAACATGAATCAGATGAATGTGACGGGG | *somA* with *Pme*I site |
| HO532 | GTTTAAACTGAAGAACCGACGGACTCATTTA | *somA* with *Pme*I site |
| HO844 | TCGAGCTCGGTACCCACTCCGAGTAGACGCCGAGGAA | *flbB* 5' UTR with pUC19 overhang |
| HO879 | TGGCCATATTATGCTGAAGTTTGTGCTCATTGAAGTGGGG | *flbB* 5’ UTR |
| HO882 | TTGACCTATAGGCCTTGAAGTGGGGGTTTTCAGGCT | *flbB* 5’ UTR with *Sfi*I site |
| HO846 | AGCATAATATGGCCACTTCTTTCTCAAGTGGACTGGGGAG | *flbB* 3' UTR with *Sfi*I site |
| HO847 | CTCTAGAGGATCCCCGACGCTGAAGAGGAGGAGGACAA | *flbB* 3' UTR with pUC19 overhang |
| HO848 | TCGAGCTCGGTACCCTTCTTGCCTCCCACCGCCTT | *stuA* 5’ UTR with pUC19 overhang |
| HO880 | TGGCCATATTATGCTTTGGTTCATGCTGCCCAGAGG | *stuA* 5’ UTR with *Sfi*I site |
| HO850 | AGCATAATATGGCCAATGCCATAGACCGGAGACAACTCG | *stuA* 3’ UTR with *Sfi*I site |
| HO851 | CTCTAGAGGATCCCCTCCACCCAGCCCTAGAGATCT | *stuA* 3’ UTR with pUC19 overhang |
| HO852 | TCGAGCTCGGTACCCGTCCTAATTTCCTGCTCGCAGCAA | *medA* 5’ UTR with pUC19 overhang |
| HO881 | TGGCCATATTATGCTAGACAGGAAGGGCTCAGACTTGGC | *medA* 5’ UTR with *Sfi*I site |
| HO854 | AGCATAATATGGCCATTCACCTTGTCTCGCATTATGGCTC | *medA* 3’ UTR with *Sfi*I site |
| HO855 | CTCTAGAGGATCCCCGTAAGGAGCCATACCTGCGCCT | *medA* 3’ UTR with pUC19 overhang |
| HO1 | CTCTTCGAAGGCTGGACTTGC | *h2A* qPCR Forward |
| HO2 | GGAGATGGCGAGGAATGATACG | *h2A* qPCR Reverse |
| HO47 | CTCACCAGGGGGTCTCAAATG | *velC* qPCR Forward |

**Table S2. Continued.**

| Primer | Sequence (5’→3’) | Description |
| --- | --- | --- |
| HO48 | CGGGGGTAGGGCTTGTATCA | *velC* qPCR Reverse |
| HO157 | AAGCCTCATGTCTGCTGGGTTC | *brlA* qPCR Forward |
| HO158 | CCGATAGTCCGGGTTGTAGTCG | *brlA* qPCR Reverse |
| HO277 | CCTGCCGTAACATTGCTTCTTG | *3G13110* qPCR Forward |
| HO278 | CACAGTCATCATCCTCCGATCC | *3G13110* qPCR Reverse |
| HO617 | AACCGGTAATGCCCAGACAGAT | *somA* qPCR Forward |
| HO618 | GTGTCCGTTCATGTCCATGTCA | *somA* qPCR Reverse |
| HO660 | GCTCTGACTCTCACTGCCTTCG | *3G00880* qPCR Forward |
| HO661 | AAGCTTGTTGACGGGAGGGTAG | *3G00880* qPCR Reverse |
| HO680 | GTACCCATCAAAAGCCGTCCTC | *medA* qPCR Forward |
| HO681 | TTCTGCATGCGAGTGAATTGAA | *medA* qPCR Reverse |
| HO682 | CTCCTGAGCACGAGTCGGAATA | *stuA* qPCR Forward |
| HO683 | CGTGGAGTCATACGTCCAGACC | *stuA* qPCR Reverse |
| HO684 | CTCCAGAGCAAGCCTATCCACA | *flbB* qPCR Forward |
| HO685 | TGCGGTACAGTTCGTGGTTCTT | *flbB* qPCR Reverse |
| HO686 | GTGACGTTGAAGGGTGTGGAAG | *flbC* qPCR Forward |
| HO687 | ACTCCTCCTCGCCACCAGATAC | *flbC* qPCR Reverse |
| HO688 | AACCTGAAGCCCTCGTTGAATC | *flbD* qPCR Forward |
| HO689 | TGGCCGAGAGACCTCTTCTCTT | *flbD* qPCR Reverse |
| HO788 | CCTATGGCCGTACCAAATGGAT | *uge3* qPCR Forward |
| HO789 | GTGGGAGTCTGTCTGGGGTCTT | *uge3* qPCR Reverse |
| HO877 | CATGATGATGCAGCAGAGGATG | *ptaB* qPCR Forward |
| HO878 | TCGACAAATGCTTGCCAATACA | *ptaB* qPCR Reverse |
| HO891 | GGTGACCAGGCTCAGCTCTCTT | *rodA* qPCR Forward |
| HO892 | TTGAAGAGACCAAGGCCCTCGG | *rodA* qPCR Reverse |
| PtaB-1 | CTGCAGGAATTCGATGTTTAAACTCAAGAGCAATTGAGACAGTT | *ptaB* 5' UTR |
| PtaB-2 | ACCTATAGGCCTGAGATTTAAATGCCTTGATGGGCAGGAAA | *ptaB* 5' UTR |
| PtaB-3 | ATAATATGGCCATCTTTTTAGAGATGGTTTCCGTTTTC | *ptaB* 3' UTR |
| PtaB-4 | ATCGATAAGCTTGATGTTTAAACTCGGCGTCTTCGCT | *ptaB* 3' UTR |
